# NEWS for Africa: adaptation and reliability of a built environment questionnaire for physical activity in seven African countries

**DOI:** 10.1186/s12966-016-0357-y

**Published:** 2016-03-08

**Authors:** Adewale L. Oyeyemi, Sandra S. Kasoma, Vincent O. Onywera, Felix Assah, Rufus A. Adedoyin, Terry L. Conway, Sarah J. Moss, Reginald Ocansey, Tracy L. Kolbe-Alexander, Kingsley K. Akinroye, Antonio Prista, Richard Larouche, Kavita A. Gavand, Kelli L. Cain, Estelle V. Lambert, Richmond Aryeetey, Clare Bartels, Mark S. Tremblay, James F. Sallis

**Affiliations:** Department of Physiotherapy, University of Maiduguri, Maiduguri, Nigeria; Department of Biochemistry and Sports Science, School of Biosciences, College of Natural Science, Makerere University, Kampala, Uganda; Department of Recreation Management and Exercise Science, Kenyatta University, Nairobi, Kenya; Department of Public Health, Faculty of Medicine and Biomedical Sciences, University of Yaoundé I, Yaoundé, Cameroon; Department of Medical Rehabilitation, Obafemi Awolowo University, Ile-Ife, Nigeria; Department of Family Medicine and Public Health, University of California, San Diego, California USA; Physical Activity, Sport and Recreation Research Focus Area, Faculty of Health Sciences, North-West University, Potchefstroom, South Africa; Active Living and Wellness Alliance Group (ALWAG), ALWAG Legacy, Nungua, Ghana; Centre for Research on Exercise, Physical Activity and Health, School of Human Movement and Nutrition Sciences, University of Queensland, Brisbane, Australia; Nigerian Heart Foundation, Lagos, Nigeria; Faculty of Physical Education and Sport Sciences, Universidade Pedagogica, Maputo, Mozambique; Children’s Hospital of Eastern Ontario Research Institute, Ottawa, Canada; Division of Exercise Science and Sports Medicine, Faculty of Health Sciences, University of Cape Town, Cape Town, South Africa; School of Public Health, University of Ghana, Legon Accra, Ghana; Department of Pediatrics, University of Ottawa, Ottawa, Canada

**Keywords:** Walkability, Active transportation, Play, Recreation, Community design, Neighborhood

## Abstract

**Background:**

Built environment and policy interventions are effective strategies for controlling the growing worldwide deaths from physical inactivity-related non-communicable diseases. To improve built environment research and develop African specific evidence, it is important to first tailor built environment measures to African contexts and assess their psychometric properties across African countries. This study reports on the adaptation and test-retest reliability of the Neighborhood Environment Walkability Scale in seven sub-Saharan African countries (NEWS-Africa).

**Methods:**

The original NEWS comprising 8 subscales measuring reported physical and social attributes of neighborhood environments was systematically adapted for Africa through extensive input from physical activity and public health researchers, built environment professionals, and residents in seven African countries: Cameroon, Ghana, Kenya, Mozambique, Nigeria, South Africa and Uganda. Cognitive testing of NEWS-Africa was conducted among diverse residents (N = 109, 50 youth [12 – 17 years] and 59 adults [22 – 67 years], 69 % from low socioeconomic status [SES] neighborhoods). NEWS-Africa was translated into local languages and evaluated for 2-week test-retest reliability in adult participants (N = 301; female = 50.2 %; age = 32.3 ± 12.9 years) purposively recruited from neighborhoods varying in walkability (high and low walkable) and SES (high and low income) and from villages in six of seven participating countries.

**Results:**

The original 67 NEWS items was expanded to 89 scores (76 individual NEWS items and 13 computed scales). Several modifications were made to individual items, and some new items were added to capture important attributes in the African environment. A new scale on personal safety was created, and the aesthetics scale was enlarged to reflect African specific characteristics. Over 95 % of all NEWS-Africa scores (items plus computed scales) demonstrated evidence of “excellent” (ICCs > .75 %) or “good” (ICCs = 0.60 to 0.74) reliability. Seven (53.8 %) of the 13 computed NEWS scales demonstrated “excellent” agreement and the other six had “good” agreement. No items or scales demonstrated "poor" reliability (ICCs < .40).

**Conclusions:**

The systematic adaptation and initial psychometric evaluation of NEWS-Africa indicates the instrument is feasible and reliable for use with adults of diverse demographic characteristics in Africa. The measure is likely to be useful for research, surveillance of built environment conditions for planning purposes, and to evaluate physical activity and policy interventions in Africa.

**Electronic supplementary material:**

The online version of this article (doi:10.1186/s12966-016-0357-y) contains supplementary material, which is available to authorized users.

## Background

Physical inactivity is a leading cause of death worldwide due to its etiologic role in multiple non-communicable diseases (NCDs) such as coronary heart disease, type 2 diabetes, and breast and colon cancers [1, 2]. However, the largest increases and burden of NCDs are in low-and middle-income countries where the understanding of strategies for increasing physical activity remains poor [3–6]. Development of strategies for reducing the growing epidemics of NCDs in low-and middle-income countries has been called urgent [7, 8]. However, for effective physical activity intervention, strategies should be evidence-based and appropriate for the country and target populations [9].

Because the determinants of physical activity are multifactorial, ecological models that recognize the centrality of environmental and policy interventions to improve health behavior have been adopted as guiding frameworks for physical activity promotion worldwide [8, 10–12]. Multiple evidence from high-and middle- income countries have linked favorable built environment designs and attributes with improved physical activity [4, 9, 13–17]. In Africa, the feasibility of conducting research on built environmental correlates of physical activity has recently been demonstrated in Nigeria, South Africa and Kenya [18–21]. However, due to the absence of contextually appropriate measures of theoretically relevant built environmental variables [22, 23], Africa remains the continent with the least research on the relation of built environmental attributes to physical activity [4, 24]. Understanding the contribution of the built environment to physical activity in Africa is important because inactivity-related NCDs are already responsible for over half of deaths and disease burden in Africa [25–27]. To improve research and develop African-specific evidence, it is important to first adapt and tailor built environment measures to African contexts, then use the adapted measures to build evidence about promising environmental solutions for African countries.

The Neighborhood Environment Walkability Scale (NEWS) is frequently used worldwide for assessing perceived attributes of the neighborhood built environment for physical activity [17, 28–32]. The NEWS is being used by the International Physical Activity and Environment Network (IPEN) in cross-country analyses of built environment and physical activity relationships. It is a reliable and valid questionnaire [28, 30, 33–35], that has been tested internationally and translated into many languages [29, 30, 35–39]. In Africa, the psychometric properties of the NEWS have been tested in Nigeria [22], with acceptable measurement properties similar to the findings in the United States, Australia and China [28, 29, 38]. However, further adaptation and evaluation of the NEWS in other African countries is needed to create a version that may be applicable for research and practice throughout the African region. Thus, the present paper reports a coordinated study of the adaptation and test-retest reliability of a modified NEWS in seven sub-Saharan African countries representing West Africa (Ghana and Nigeria), Southern Africa (Mozambique and Republic of South Africa), East Africa (Kenya and Uganda) and Central Africa (Cameroon).

## Methods

### Design

This study was conducted in three stages: (1) development and adaptation of the NEWS that included input from researchers, local experts, and residents; (2) pilot study including cognitive testing and translations; (3) test-retest reliability assessment.

### Stage 1: Development and adaptation

#### Methods and results

The 67-item original NEWS instrument was developed in the United State to obtain residents’ perceptions of neighborhood design features proposed in the transportation and urban planning literature to be related to physical activity [28, 40]. Items on the original NEWS were a priori grouped into 8 subscales to assess the underlying constructs of residential density, proximity to stores and facilities, perceived access to destinations, street connectivity, facilities for walking and cycling, aesthetics, and safety from traffic and crime [28]. To develop a version of the NEWS for use throughout sub-Saharan African countries, 16 physical activity and public health researchers from 7 countries in Africa (11 members), United States (3 members) and Canada (2 members) convened a meeting in Kenya in 2013 with a goal to draft a NEWS-Africa questionnaire that could be applicable across the lifespan (youth, adults and older adults). This committee carefully reviewed all sections of the original NEWS instrument, including additional items from the NEWS-Youth [41] and NEWS for older adults [31]. The committee discussed the relevance of each item for Africa (with the goal of retaining as many as possible to enhance comparability with results from other regions), modifications of current items, and additions of items needed to reflect African urban and rural environments and cultures. The committee received additional inputs from local professionals in Cameroon, Ghana, Kenya, Nigeria, South Africa and Uganda, who had backgrounds in diverse fields such as community design, transportation, parks, and public health advocacy. Information also was solicited from community residents, recruited from women's empowerment groups (frequent walkers) and parents and youth who used the environment for physical activity. The community residents were interviewed on the relevance of the neighborhood environment features assessed by NEWS to physical activity for transport and leisure purposes in their neighborhoods. They were informed that the questionnaire was originally designed to assess attributes of the neighborhood environment that are important for physical activity in developed countries, and were encouraged to provide critical feedback on the relevance of these neighborhood environment attributes to Africa and their suggestions for improving the items.

Key outputs of the adaptation process were a draft modified NEWS agreed to by the committee, based on modification and clarification of words and concepts on the original 67 items NEWS instrument to the African context, inclusion of 14 new items to reflect the African context, and recognition of the need to simplify the survey using terms familiar with local people (see Additional file 1 and Table 2). Specifically, the 6 items on residential density were reduced to a single unweighted item, and the response scale was refined to include common housing patterns in urban and rural areas of Africa. The response scale was also reordered from the lowest to the highest housing density. Common destinations like places for farming, hunting, collecting firewood, fetching water, king’s palace, and village square were added to the section on land-use mix. In addition to the original questions on roads and pedestrian infrastructures, items were added to focus on unpaved walking/foot paths to reflect the stage of infrastructure development in most African countries. To simplify understanding, clarification about roads, routes and pathways were provided to indicate whether roads were formal/official routes for cars (paved or unpaved) with or without pedestrian facilities, versus informal (unofficial) pathways, shortcuts and foot paths where people could walk or bicycle and were not primarily meant for cars.

### Stage 2: Pilot study (Cognitive testing and translations)

#### Methods and results

The draft modified NEWS produced through the above process was subjected to two rounds of revisions that involved cognitive testing in six countries (Cameroon, Ghana, Kenya, Nigeria, South Africa and Uganda) and consensus discussion among members of the committee. Adult and adolescent community residents in each country who were able to walk without assistance and could communicate verbally, with no diagnosis of cognitive impairment participated in cognitive pretesting of the modified NEWS. Each participant in the pilot study was interviewed for their understanding of the words in the questionnaire, clarity of each item and response options, and their suggestions for improvement (similarly for adults and youth). The participants were asked if any question made them feel uncomfortable and if any relevant environmental attribute in the local context was not included in the questionnaire. All participants were encouraged to verbalize their thought process while providing responses to the items.

Participants for the cognitive evaluation were recruited mainly from middle to low socioeconomic status (SES) areas, with the exception of Kenya that included some high SES residents. Cognitive interviews were conducted only among adults in South Africa (n = 30, age = 22 – 57 years) and Cameroon (n = 6, age = 24 – 45 years), only among youth in Kenya (n = 30, age = 10-12 years), but among both adults and youth in Nigeria (n = 2 adults, age = 42 – 67 years; n = 4 youth, age = 13 – 16 years), Ghana (n = 6 adults, age = 20 – 45 years; n = 6 youth age = 12 – 15 years) and Uganda (n = 15 adults, age = 22 – 52 years; n = 10 youth, age = 15 – 17 years). The mean ages of the youth and adult participants in the cognitive testing were 13.1 ± 2.3 years and 37.2 ± 10.8 years, respectively. The most problematic items on the questionnaire identified by the participants were those on residential density, land use mix – diversity (stores, facilities, and other things in the neighborhood), land use mix – access (access to services and places), and infrastructure and safety for walking and cycling (Additional file 2). The question on residential density was difficult to understand by some adults in South Africa and Uganda, but mostly difficult to understand by youth in Nigeria without detailed description and explanation by the interviewers. For the section on stores, facilities, and other things in the neighborhood, the concept of time taken to walk to destinations was difficult for some adult participants in South Africa and some youth in Kenya and Uganda, but this was not a problem for adults and youth in Nigeria and Ghana. The section on access to services and places was mostly problematic for youth in Kenya and Nigeria because the response options ranging from “strongly disagree” to “strongly agree” were difficult to comprehend. While this section was not difficult for participants in South Africa (adults), Cameroon (adults) and Ghana (adults and youth), the opening phrase “It is easy…” for most of the items in the section was ambiguous and confusing to most participants in Uganda as they could not determine whether “easy” was in terms of physical ability, physical terrain to traverse or distance to walk. The two sections on road and walking paths and places for walking, cycling and playing posed some difficulties to youth from low SES areas in Kenya, and some participants (adults and youth) in Uganda. Five items that focused on not commonly available pedestrian infrastructures were difficult to comprehend by youth participants in Nigeria. However, most of the difficult items identified during the cognitive testing were resolved by wording changes, inclusion of a “not applicable” response option to some items identified by some participants as not available in their neighborhood and explanation by interviewers. The summary of results from the cognitive study are provided in Additional file 2.

Through consensus discussion by the committee, results of the cognitive study were used to improve the modified NEWS, and to produce a harmonized version (hereafter called NEWS-Africa). The key outputs from the cognitive study were the identification of the need to recommend the NEWS-Africa as an interview-administered survey rather than self-administered, and the development of the NEWS-Africa interview manual (Additional file 3) and accompanying photos (Additional file 4) to standardize interview strategies and improve quality of data across countries. The interview manual and photos for NEWS-Africa were developed through consensus discussion by the committee to guide interviewers on how to probe, clarify concepts, and facilitate better understanding of difficult items.

Next, the harmonized version of the NEWS-Africa and the interviewer manual were translated into local or dominant languages in Cameroon (French), Ghana (Twi and Krobo), Kenya (Swahili), Mozambique (Portuguese), Nigeria (Yoruba), South Africa (Xhosa) and Uganda (Luganda) by a knowledgeable bilingual person in each of the countries. Translators were instructed to use simple terms understood by people from a wide range of education levels. For each country, the translation was back-translated into English by someone who was bilingual and not familiar with the project but could be representative of eventual study participants. To ensure that underlying concepts assessed by the NEWS-Africa instrument had not been compromised during the translation process, the back-translated version in each of the countries was reviewed by a team that included the translator, the country lead investigator and two international experts who were familiar with the constructs of the original NEWS instrument and part of the development of the NEWS-Africa questionnaire. Consensus discussions among all investigators were thereafter used to produce the final version of NEWS-Africa that was used for psychometric evaluations in all participating countries.

The final NEWS-Africa survey produced through these processes consisted of 76 individual items that assessed the following perceived environmental characteristics: residential density (1 item); proximity to non-residential land uses (land use mix - diversity) (27 items); ease of access to nonresidential uses (land use mix - access) (7 items); street connectivity (5 items); infrastructure and safety for walking and cycling (12 items); aesthetics (8 items); traffic safety (6 items); safety from crime (4 items), personal safety (3 items) and child-related questions on stranger danger (3 items). Two scales (destinations scale [21 items] and recreation facility scale [4 items]) were derived from the section on land use mix – diversity, and 3 additional scales (sidewalks scale [5 items], street crossing scale [4 items] and path infrastructure scale [2 items]) were computed from the section on infrastructure and safety for walking and cycling (see Additional file 1 and Table 2).

### Stage 3: Test-retest reliability

#### Methods

Adult participants from six African countries (Cameroon, Ghana, Mozambique, Nigeria, South Africa and Uganda) were purposively recruited for the test-retest reliability study. Kenya was not included in stage 3 because only participants below 13 years old had been recruited. Participants in the reliability study were selected from neighborhood areas that varied in SES (high/low income) and transport related walkability (high/low walkable), with some recruited from rural villages. While information on neighborhood area SES were obtained from appropriate government ministries, departments or agencies in each country and based on previous experience from built environment studies in Africa [18–20, 42], neighborhood walkability classification was based on the criteria from the transportation literature [38, 40]. According to the transportation literature, high-walkable neighborhoods are characterized by a high residential density, high concentration of non-residential land uses (retail shops, local markets and places of worship) and streets with short block length with many alternative routes to destinations. Low-walkable neighborhoods are characterized by low residential density (predominantly separate, single family homes), few non-residential land uses, and streets with longer block length with fewer alternative routes to destinations [38, 40]. Neighborhoods fitting these general criteria were identified based on local knowledge, with the goal of ensuring variability of environments.

Within identified neighborhoods, participants were selected using “door-to-door” recruitment procedures. Only one adult (18 + years) per household was eligible to participate in the study. Participants deemed ineligible for the psychometric study were those in group living establishments (e.g., health-care facilities, dormitories, military school barracks), below 18 years of age, who had not lived within the identified neighborhood for at least 6 months or were unwilling to complete the interview survey in English or a local language. A modest incentive (monetary or gift) was provided in some countries for completing the re-test assessments that were conducted approximately 2 weeks after the initial survey was completed. All participants provided informed consent, and the study was approved by the appropriate ethical review committee of the principal investigator’s institution in each country.

Demographic characteristics of age, sex, education, income, access to automobile in the household, bicycle ownership, and height and weight were obtained on the first day of contact with the participants. Participants reported their height and weight in two countries, and these variables were directly measured in Nigeria, Mozambique, South Africa and Uganda. BMI (weight/square of height) derived from self-report and direct measurements are highly correlated, and are both used as proxy measure of adiposity in large scale studies [43].

#### Data analysis

Descriptive analyses (e.g., means and SDs for continuous variables or frequencies and percentages for categorical variables) were computed for all measures (see Table 1 for participant characteristics and Table 2 for NEWS-Africa measures completed at the initial assessment). To assess test-retest reliability of the NEWS-Africa items and computed scales, intraclass correlation coefficients (ICCs) were computed using SPSS Version 21. The SPSS Scale/Reliability procedure was used to compute ICCs using the two way-mixed (absolute) model for single measures. As an additional check on the stability of measures, ANOVA for repeated measures was performed to test for significant mean differences in items and scales between the initial and re-test assessments. Because of the large number of tests (89 items and scales), the Bonferroni correction was applied to adjust the p-value for significance to be p < 0.00056 [i.e., “experiment-wide” p-value = (.05/89) = 0.000562)].

**Table 1:** Descriptive characteristics for the test-retest reliability sample (*n* = 301)

Characteristic		N (%)
Country		
	Cameroon	43 (14.3)
	Ghana	39 (13.0)
	Mozambique	64 (21.3)
	Nigeria	72 (23.9)
	South Africa	40 (13.3)
	Uganda	43 (14.3)
Region		
	Urban	240 (79.7)
	Suburban/Periurban	32 (10.6)
	Rural	29 (9.6)
Study design quadrant		
	High walkability/Low Income	63 (20.9)
	High walkability/High Income	46 (15.3)
	Low walkability/Low Income	26 (8.6)
	Low walkability/High Income	102 (33.9)
	Missing (%) ^a^	64 (21.3)
Gender – Female		151 (50.2)
Marital Status – Married or living with partner		131 (43.5)
Education		
	No formal education	7 (2.3)
	Primary school	13 (4.3)
	Some high school	61 (20.3)
	Completed high school	75 (24.9)
	Diploma/higher diploma	27 (9.0)
	Bachelor’s degree	92 (30.6)
	Graduate/professional degree	26 (8.6)
Motorized vehicles per household		
	None	130 (43.3)
	One	90 (30.0)
	Two	54 (18.0)
	Three or more	26 (8.7)
		Mean (SD)
Age (years)		32.3 (12.9)
Adults in Household		3.6 (2.9)
Youth in Household		2.4 (2.3)
Number of months lived in house		102.9 (112.4)
Body Mass Index (BMI, kg/m2))		25.0 (5.0)

^a^ Mozambique was unable to select participants by quadrant.

**Table 2:** NEWS-Africa Item and Scale Descriptives and Test-Retest Reliabilities (*n* = 301)

Item/Scale	Range	Test-Retest Reliability: ICC (95 % CI)	Mean‡	(SD)‡
A. Types of residences				
Types of residences in your neighborhood (not weighted)*	1-6	0.80 (0.75, 0.83)	2.88	1.69
B. Stores, facilities and other things in your neighborhood				
B1. Kiosk/corner store/ small grocery	1-5	0.73 (0.67, 0.78)	4.22	1.08
B2. Supermarket	1-5	0.73 (0.66, 0.78)	2.80	1.32
B3. Fruit/vegetable market	1-5	0.73 (0.67, 0.78)	2.78	1.36
B4. Fast food restaurant	1-5	0.82 (0.78, 0.86)	3.06	1.49
B5. Non-fast food restaurant	1-5	0.71 (0.65, 0.77)	2.82	1.52
B6. Pub or bar*	1-5	0.69 (0.62, 0.74)	3.51	1.43
B7. Cinema/theatre*	1-5	0.62 (0.54, 0.69)	1.55	1.04
B8. Place of worship/ faith centre**	1-5	0.67 (0.60, 0.73)	2.84	1.41
B9. Computer/cell kiosks*	1-5	0.75 (0.69, 0.79)	3.07	1.50
B10. Library	1-5	0.58 (0.49, 0.66)	1.68	1.10
B11. Any school	1-5	0.69 (0.63, 0.75)	3.12	1.47
B12. Your workplace or school	1-5	0.72 (0.65, 0.77)	2.05	1.41
B13. Book store	1-5	0.69 (0.61, 0.74)	1.98	1.30
B14. Health clinic/ hospital*	1-5	0.69 (0.62, 0.74)	2.44	1.33
B15. Pharmacy/ chemist	1-5	0.74 (0.68, 0.79)	3.05	1.42
B16. Salon/ barber shop	1-5	0.66 (0.58, 0.72)	3.72	1.28
B17. Clothing store	1-5	0.71 (0.65, 0.77)	2.48	1.45
B18. Electronics shop*	1-5	0.74 (0.68, 0.79)	2.43	1.45
B19. Public bus or train stop	1-5	0.70 (0.64, 0.76)	2.76	1.53
B20. Taxi motorbike stop*	1-5	0.68 (0.60, 0.74)	3.47	1.45
B21. Sports field or court*	1-5	0.69 (0.62, 0.74)	2.47	1.40
B22. Other outdoor recreation space	1-5	0.70 (0.63, 0.75)	2.14	1.42
B23. Other indoor recreation facilities	1-5	0.71 (0.64, 0.77)	2.03	1.27
B24. Dance/ martial arts*	1-5	0.63 (0.55, 0.70)	1.59	1.17
B25. Tap/well, pond, river or stream for fresh water**	1-5	0.50 (0.41, 0.58)	4.36	1.25
B26. Farm**	1-5	0.71 (0.64, 0.76)	1.86	1.45
B27. Places for hunting/ collecting firewood**	1-5	0.76 (0.70, 0.81)	1.57	1.19
Destinations scale – mean of items 1-20 and 25	1.14-4.75	0.79 (0.74, 0.83)	2.90	0.74
Recreation scale – mean of items 21-24	1-5	0.74 (0.68, 0.79)	2.10	1.01
C. Access to services and places				
C1. Stores are within easy walking distance of my house	1-4	0.68 (0.61, 0.74)	3.20	1.04
C2. There are many places to go, such as food markets and restaurants, within easy walking distance of my house	1-4	0.73 (0.68, 0.78)	2.80	1.12
C3. It is easy to walk to a transit/transport stop from my house	1-4	0.68 (0.61, 0.73)	3.06	1.06
C4. It is easy to walk to an outdoor recreation place space from my house*	1-4	0.62 (0.55, 0.69)	2.48	1.16
C5. It is easy to walk to an indoor recreation facility from my house*	1-4	0.62 (0.54, 0.69)	2.20	1.09
C6. Places to get essential supplies like water and firewood are within easy walking distance of my house**	1-4	0.63 (0.56, 0.69)	3.01	1.10
C7. There are gathering places within easy distance of my house*	1-4	0.66 (0.59, 0.72)	2.77	1.04
Access to services scale – mean of items 1-7	1-4	0.71 (0.64, 0.76)	2.79	0.62
D. Roads and walking paths in my neighborhood				
D1. The distance to walk to the closest next street in my neighborhood is usually short	1-4	0.52 (0.43, 0.60)	3.27	0.96
D2. There are many alternative roads for getting from place to place in my neighborhood	1-4	0.54 (0.46, 0.62)	3.26	0.95
D3. There are many unofficial routes connecting places in my area**	1-4	0.62 (0.54, 0.68)	2.86	1.09
D4. There are many shortcuts such as foot paths between roads in my area*	1-4	0.64 (0.57, 0.71)	2.80	1.10
D5. Some roads or walking/foot paths in my area are blocked by gates or barriers*	1-4	0.68 (0.61, 0.73)	2.76	1.14
Roads and walking paths scale – mean of items 1-5 (after recodes)	1.20-4	0.60 (0.53, 0.67)	2.99	0.54
E. Places for walking, cycling and playing				
E1. There are formally provided sidewalks on most of the roads in my neighborhood	1-4	0.75 (0.69, 0.79)	2.00	1.17
E2. The sidewalks in my neighborhood are well maintained*	1-4	0.71 (0.65, 0.76)	1.77	1.08
E3. The sidewalks in my neighborhood are often blocked by merchandise, construction materials, etc.**	1-4	0.68 (0.61, 0.73)	2.37	1.24
E4. Sidewalks are separated from the road in my neighborhood by parked cars…	1-4	0.61 (0.53, 0.68)	1.80	1.08
E5. There is a grass/dirt strip that separates the road from the sidewalks in my neighborhood	1-4	0.74 (0.69, 0.79)	1.57	1.01
E6. There are signals or crosswalks / zebra crossings to help walkers cross the busy roads in my neighborhood	1-4	0.74 (0.68, 0.79)	1.72	1.09
E7. There are curb ramps that go from sidewalk level to road level at road crossings in my neighborhood to assist the elderly…*	1-4	0.72 (0.66, 0.77)	1.60	1.02
E8. There is enough time for people on foot to cross the road at crossing points/junctions with traffic lights, signals or robots*	1-4	0.68 (0.61, 0.73)	1.93	1.14
E9. In my neighborhood there are busy roads that are dangerous to cross*	1-4	0.60 (0.52, 0.67)	2.58	1.19
E10. There are informal places for people to walk in my neighborhood**	1-4	0.60 (0.52, 0.67)	2.69	1.13
E11. The walk/foot paths in my neighborhood are generally of good quality…**	1-4	0.60 (0.53, 0.67)	2.13	1.14
E12. There are designated or marked places to bicycle…*	1-4	0.60 (0.52, 0.67)	1.60	0.99
Sidewalk scale – mean of items 1-5	1-4	0.76 (0.71, 0.80)	1.90	0.73
Crossing scale – mean of items 6-9	1-4	0.70 (0.64, 0.75)	1.96	0.67
Path infrastructure scale – mean of items 10-11	1-4	0.60 (0.52, 0.67)	2.41	0.88
Places for walking, cycling, and playing overall scale – mean of all items in Section E	1.08 – 3.92	0.80 (0.75, 0.83)	1.98	0.56
F. Neighborhood Surroundings				
F1. There are trees along the roads in my neighborhood	1-4	0.74 (0.68, 0.78)	2.34	1.24
F2. My neighborhood is clean and free of litter, garbage or stagnant water*	1-4	0.68 (0.61, 0.73)	2.60	1.10
F3. My neighborhood is free from bad smells and odors**	1-4	0.60 (0.52, 0.67)	2.77	1.10
F4. There are beautiful natural sights/ views in my neighborhood	1-4	0.75 (0.70, 0.80)	2.47	1.12
F5. There are attractive buildings/ houses in my neighborhood	1-4	0.74 (0.69, 0.79)	2.82	1.02
F6. My neighborhood is generally free of unpleasant noises like highways, factories, trains, bars…**	1-4	0.62 (0.54, 0.69)	2.80	1.12
F7. My neighborhood is generally free of noticeable pollution and dust, such as from traffic or factories*	1-4	0.63 (0.56, 0.69)	2.75	1.05
F8. There are many pleasant natural sounds in my neighborhood such as from birds**	1-4	0.67 (0.60, 0.73)	2.66	1.06
Neighborhood surroundings scale - mean of items 1-8	1-4	0.78 (0.73, 0.82)	2.65	0.66
G. Safety from Traffic				
G1. There is so much traffic along nearby roads that it is difficult or unpleasant to walk or play in my neighborhood	1-4	0.67 (0.60, 0.73)	2.68	1.09
G2. The speed of traffic on most nearby roads in my neighborhood is usually slow	1-4	0.63 (0.55, 0.69)	2.65	1.06
G3. Most drivers exceed the speed limits in my neighborhood	1-4	0.62 (0.55, 0.69)	2.54	1.09
G4. Walking or playing is dangerous in my neighborhood because of careless or aggressive driving**	1-4	0.66 (0.59, 0.72)	2.69	1.06
G5. It could be dangerous to ride a bicycle in or near my neighborhood because of speed of traffic*	1-4	0.70 (0.64, 0.75)	2.93	1.13
G6. I am worried about playing or walking in my neighborhood and local streets because I am afraid of being injured by a car*	1-4	0.77 (0.68, 0.83)	2.09	1.09
Traffic scale - mean of items G1-5 and PC4	1-4	0.75 (0.70, 0.80)	2.65	0.68
H. Safety from Crime				
H1. There is a lot of crime in my neighborhood.	1-4	0.79 (0.73, 0.83)	2.89	1.07
H2. There is too much crime in my neighborhood to go outside for walks or play during the day.	1-4	0.71 (0.65, 0.76)	3.37	0.84
H3. There is too much crime in my neighborhood to go outside for walks or play at night.	1-4	0.79 (0.74, 0.82)	2.95	1.06
H4. There are groups of people or gangs in my neighborhood who make me feel threatened when I go out.**	1-4	0.73 (0.67, 0.78)	3.04	1.06
Crime scale - Mean of items 1-4 after reverse coding	1-4	0.82 (0.78, 0.86)	3.06	0.82
I. Personal Safety				
I1. I see and can talk to people when I am walking in my neighborhood.*	1-4	0.63 (0.55, 0.69)	3.27	0.89
I2. There are stray dogs or dangerous animals that scare me in my neighborhood.**	1-4	0.68 (0.61, 0.74)	2.77	1.15
I3. The roads in my neighborhood are well lit at night.	1-4	0.70 (0.64, 0.75)	2.33	1.19
Personal Safety Scale - Mean of I1-2 and E9	1-4	0.67 (0.60, 0.73)	2.80	0.61
J. Stranger Danger				
PC1. I am worried about *my child *playing or being outside alone *or with friends *around my house (e.g. yard, driveway, apartment common area), because I am afraid of them being taken or hurt by a stranger.*	1-4	0.73 (0.65, 0.80)	2.34	1.13
PC2. I am worried about *my child* playing or walking alone or with friends in my neighborhood and local streets because I am afraid of them being taken or hurt by a stranger.*	1-4	0.77 (0.69, 0.83)	2.24	1.16
PC3. I am worried about *my child* being alone or with friends in a local or nearby park because I am afraid of them being taken or hurt by a stranger*	1-4	0.78 (0.70, 0.84)	2.30	1.13
Stranger Danger Scale - Mean of parent-reported Items 1-3	1-4	0.79 (0.72, 0.84)	2.29	1.03

* Modified items to reflect African context

**New items added specifically for NEWS-Africa.

‡ Mean and standard deviation (SD) computed from the Time 1 survey.

A variety of numeric definitions and adjectival descriptors have been used to classify measures of inter-rater agreement using kappas for categorical variables and ICCs for test-retest of continuous measures [44–47]. For this study, Cicchetti’s [47] numeric ranges and descriptors were used. ICCs were classified as indicating test-retest reliability that was: ‘excellent’ (ICC ≥ .75), 'good' (.60 - .74), 'fair' (.40 - .59), and 'poor' (< .40).

## Results

A total of 301 participants completed the NEWS-Africa survey at two time points approximately two weeks apart. Descriptive characteristics are summarized in Table 1. A range of 39 to 72 participants from each of 6 African nations completed test-retest surveys. Participants lived predominantly in urban areas (79.7 %) that were relatively balanced between neighborhoods representing “lower walkability” (42.5 %) and “higher walkability” (36.2 %); walkability for the other 21.3 % was unknown (all from Mozambique, which was unable to select participants based on the walkability/SES criteria). Somewhat more “higher income” (49.2 %) versus “lower income” (29.6 %) neighborhoods were represented, with the rest unknown.

The sample averaged 32.3 years of age (range 18-85 years). Participants included about half women and men, with 43.5 % married or living with a partner. The sample included a full range of educational attainment with 26.9 % not having completed high school, 33.9 % completing high school or having a diploma, and 39.2 % having a bachelor’s degree or higher. Regarding weight status, based on BMI, 57.6 % of participants were in the normal range (BMI <25.0), 29.2 % were in the overweight range (BMI = 25.0-29.9), and 13.2 % were in the obese range (BMI ≥30.0).

The average time participants had lived in the house was more than 8.5 years. Just over 43 % reported living in households with no motorized vehicles available.

All NEWS-Africa survey items and the computed scales (shown at end of each section of items used in the scale computations) are provided in Table 2. Also shown are the ICCs (and 95 % confidence intervals) indicating test-retest reliabilities for all individual items and scales. Only Time 1 means and standard deviations are tabled because ANOVA results comparing test-retest means indicated very few significant differences between surveys (only 3 of 89 comparisons were significant).

The average ICC across all 89 comparisons was 0.69 (SD = 0.07, range 0.50-0.82). For the 76 individual NEWS items, the average ICC was 0.68 (SD = 0.06, range 0.50-0.82). Average ICC was slightly higher for the 13 computed scales, with the mean scale ICC = 0.73 (SD = 0.07, range 0.60-0.82). Using Cicchetti’s [47] classifications, Table 3 shows the number (and percentage) of individual items, computed scales, and total combined measures of ICCs falling into categories reflecting “excellent”, “good”, and “fair” agreement (none fell in the “poor” range) between responses in the test and retest surveys. Over 95 % of all NEWS measures (individual items plus computed scales) fell in the “excellent” or “good” categories. Over half (*n* = 7, 53.8 %) of the computed NEWS scales fell in the “excellent” agreement category, and the remaining scales (*n* = 6, 46.2 %) fell in the “good” category.

**Table 3:** NEWS-Africa ICC reliability classifications^a^ of individual items, computed scales, and combined total

ICC Classifications^a^	"Excellent" agreement	"Good" agreement	"Fair" agreement	
	(ICCs ≥ 0.75)	(ICC = 0.60 to 0.74)	(ICC = 0.40 to 0.59)	Total
	N (%)	N (%)	N (%)	N (%)
Individual items	11 (14.5)	61 (80.2)	4 (5.3)	76 (100.0)
Scales (means of items)	7 (53.8)	6 (46.2)	0 (0.0)	13 (100.0)
Total (items + scales)	18 (20.2)	67 (75.3)	4 (4.5)	89 (100.0)
^a^ Cicchetti (2001)				

## Discussion

To evaluate the role of built environments in physical activity and risk for NCDs, it is necessary to have built environment measures that are appropriate to the context. Africa is the continent with the fewest studies of built environments and physical activity [4, 24], and more studies are needed because NCDs are responsible for over half of deaths and disease burden in Africa [25–27]. A widely used built environment self-report measure was systematically adapted for the African context and pilot tested in seven sub-Saharan African countries. The resulting NEWS-Africa instrument was evaluated for test-retest reliability with adults in six countries. Over 95 % of items and scales were found to have "excellent" or "good" reliabilities, with most scales in the "excellent" category and most items in the "good" category. None of the items or scales were in the "poor" category. This initial psychometric evaluation of NEWS-Africa indicates the instrument is feasible and reliable for use with adults of diverse demographic characteristics in six African countries.

The systematic adaptation process confirmed the need for an Africa-specific built environment measure. Based on input from physical activity researchers, professionals with built environment and public health expertise, and residents of seven countries, many modifications were made to the original instrument that was developed in the United States [28]. Though the goal was to retain as many items and constructs as possible to support comparison of results across countries, some of the scales had to be transformed. One example was residential density, which was dramatically changed to a single item with response options enlarged to include rural areas and densely packed slums. The original 67 NEWS items was expanded to 76 individual items, plus 13 computed scales, to include important attributes in the African context. Many modifications were made to the list of common destinations, and several items about informal roads and walking paths were introduced. A new scale on personal safety was created from disparate items related to dangerous animals, lighting at night, and seeing people walking in the neighborhood. The aesthetics scale was enlarged to represent sounds/noise and smells that could affect the experience of being active in the neighborhood. The pilot study led to important decisions that NEWS-Africa should be interviewer-administered with accompany user manual, and photographs were needed to illustrate some concepts. The extensive adaptation and development process was somewhat validated by feedback from the cognitive testing that led to few wording changes or new items.

NEWS-Africa was developed with different goals in mind than previous versions of the NEWS. Initially, the NEWS was developed for adults [28], then modified for youth [41] and older adults [31]. The NEWS-Africa development committee had interest and expertise across the entire age spectrum, so the adaptation was designed to be applicable to all ages (ages 12+ years). The original NEWS was designed for use in urban and suburban environments, but the large rural population in Africa made it imperative to broaden the scope of the measure, even though the literature on assessment of rural environments in relation to physical activity is limited. Adaptations for rural settings drew mainly on local knowledge from informants, supplemented by published studies [48, 49]. Another goal was to produce a measure that could be used throughout sub-Saharan Africa, because use of a common measure in the region could accelerate understanding better than use of multiple instruments whose results could not be compared. Active engagement of leading researchers from several regions of Africa helped produce an instrument that incorporated regional differences. Present results demonstrated the measure has wide applicability in several countries and several local or dominant languages in Africa, hopefully leading to common use throughout the region.

Analyses of specific items and multi-item scales supported the psychometric performance of NEWS-Africa among adults, and the effectiveness of the development and adaptation process. No item had "poor" reliability, so no item is recommended for deletion. Given the educational diversity of the sample and that almost 27 % did not complete high school, the strong results for test-retest reliability indicated the interviewer-administration protocol worked well. The same scales were retained from the original NEWS, and the majority had "excellent" reliability. One new scale on personal safety was developed for NEWS-Africa. Given the many differences in African environments compared to other regions, it may be useful to explore other options for combining items into scales. For example, scales specific to rural settings may be useful for some purposes. A next step in the evaluation is to examine construct validity through correlations with physical activity, weight status, and other health-related outcomes.

Strengths of the study included the adaptation process that engaged researchers from multiple African countries and solicited input from a wide range of informants including built environment professionals and community residents. The goals of broad applicability across sub-Saharan African countries, age groups, and the urban-rural spectrum affected many decisions about the scale. Cognitive testing and pilot testing led to important changes in procedures and materials that likely enhanced the quality of the measure, as reflected in the test-retest reliability results. Conducting coordinated psychometric studies in multiple countries is not common, but it is important for promoting widespread use of a common measure.

The present study had some limitations. The modest sample size, non-probability nature of the sample and evaluation of test-retest reliability of the adapted NEWS among adults limit generalizability of the findings. It is important for future studies to test the psychometric properties of NEWS-Africa in samples of youth and older adults to determine its applicability across the entire age spectrum in Africa. We were also unable to compare high- and low- walkable communities in all countries, on the basis of the current definition of walkability. The lack of testing of internal consistency of NEWS-Africa items could be considered another limitation. However, the authors believe that internal consistency should not be considered an indicator of quality of environmental measures, as it is for psychological measures. If sidewalk quality is not correlated with a grass/dirt strip separating the sidewalk from the street, it does not mean the scale is of poor quality. They are both considered indicators of a favorable environment for walking. Past uses of factor analysis to produce internally consistent scales led to scales that were difficult to interpret [50]. However, to create scales for NEWS-Africa that may be better indicators of African environments it would be useful to explore the construct and predictive validity of the questionnaire in future studies. “Ground truthing” studies using GIS, in terms of actual distances to amenities, traffic and crime may provide additional insights into factors related to the perceived environment, which may impact on physical activity. Certainly the length of NEWS-Africa is a barrier/limitation to widespread use, and in large studies. A goal of future studies should be to shorten the instrument by removing items that are shown to be unrelated to outcomes of interest in Africa.

## Conclusions

In summary, NEWS-Africa was systematically designed to measure built and social environment attributes with relevance to physical activity but also tailored to the African context through extensive local input and pilot testing. The resulting items and scales performed very well as evidenced by over 95 % being categorized as "excellent" or "good" reliability. Because the instrument was evaluated among a diverse sample of adults from six countries and in multiple languages, NEWS-Africa can be recommended for further use throughout sub-Saharan Africa. The measure is likely to be useful for research, surveillance of built environment conditions for planning purposes, and to evaluate policy interventions.

## Additional files

Additional file 1: News-Africa survey (PDF 441 kb) Additional file 2: Summary of results of cognitive testing of NEWS-Africa survey (DOC 42 kb) Additional file 3: Interview manual for NEWS-Africa survey (PDF 375 kb) Additional file 4: Picture guide for NEWS-Africa survey (PDF 1318 kb)
