# Supplementary material for: NEWS for Africa: adaptation and reliability of a built environment questionnaire for physical activity in seven African countries
Source: Int J Behav Nutr Phys Act. 2016 Mar 8;13:33. doi: 10.1186/s12966-016-0357-y (PMC4782343; doi:10.1186/s12966-016-0357-y)
Supplement: Additional file 1: — News-Africa survey (PDF 441 kb) [file 12966_2016_357_MOESM1_ESM.pdf]

NEWS-AFRICA SURVEY

This questionnaire has been used all over the world to understand more about where people live and how this may impact their ability and willingness to walk, move about, and play. This version has been adapted for use in sub-Saharan Africa countries for people aged 12-100 years. *Not all questions may apply to you,* but please try to answer all questions to the best of your ability.

SECTION A: DEMOGRAPHIC INFORMATION

Please respond to these questions about yourself.

1. What is your age?__________________(years old)

2. What is your sex?
   1. ☐ Male
   2. ☐ Female

3. What is your marital status?
   1. ☐ Married
   2. ☐ Living with partner
   3. ☐ Single
   4. ☐ Widowed/divorced/separated
   5. ☐ Not applicable for youth 17 and younger

4. What is your highest level of education?
   1. ☐ No formal schooling
   2. ☐ Primary school
   3. ☐ Some high school
   4. ☐ Completed high school
   5. ☐ Diploma/Higher Diploma
   6. ☐ Bachelor's degree
   7. ☐ Graduate (Masters/PhD)/professional degree

5. How many functioning motorized transport/vehicles (cars, trucks, motorcycles/bikes, tricycles) are available for use in your household?
   1. ☐ None
   2. ☐ One
   3. ☐ Two
   4. ☐ Three or more

6. How often do you use personal or private motorized transport (cars, trucks, motorcycles/bikes, tricycles) in a week? This can be your vehicle or someone else's, but not public transport.
   1. ☐ Not at all or Less than once a week
   2. ☐ A few times a week
   3. ☐ Most days of the week
   4. ☐ Everyday

7. What is the **COMBINED** monthly income for your household? ***Modify as needed to reflect specific income cut-points in your country***

1. ☐ Less than $200
2. ☐ Between $200-599
3. ☐ Between $600-999
4. ☐ Between $1,000-$1,999
5. ☐ Between $2,000-$3,000
6. ☐ Between $3,000-$5,000
7. ☐ More than $5,000

8. How many adults (aged 18 and above) live in your household most of the time? ____ adults

9. How many youth (aged 0-17 years) live in your household most of the time? _____ youth

10. How long have you lived in your present address (house)? ______ years and ______ months

11. What is your height: _____ Metres ***modify as needed to reflect the desired unit of measurement.***

12. What is your weight: _______ Kg ***modify as needed to reflect the desired unit of measurement.***

SECTION B: QUESTIONS ABOUT YOUR NEIGHBORHOOD

*We would like to find out more information about what you perceive or think and how you feel about your neighborhood. By neighborhood we mean **ALL** the area that you could walk to in **10-15 minutes from your house** (within approximately one kilometer or half a mile of your house). Please check the answer that best applies to you and your neighborhood.*

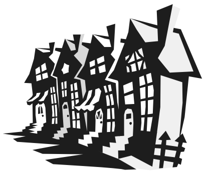

A. Types of residences in your neighborhood

Please put a check mark (√) in the box beside the answer that best applies to you and your neighborhood (please select only one answer).

1. What is the main type of housing in your immediate neighborhood?
    1. ☐ Very few residential buildings/dwellings within 2-5 min walk of my house
    2. ☐ Detached or semi-detached single-family houses with space/garden
    3. ☐ Attached (row) housing, apartment blocks/flats or multi-family housing with 2-5 stories.
    4. ☐ Multiple apartment blocks/flats of 6 stories or more, with large spaces between buildings.
    5. ☐ Multiple apartment blocks/flats of 6 stories or more, with very little space between buildings.
    6. ☐ Very densely packed small houses (1-story homes, including informal settlements and slums)

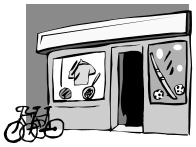

B. Stores, facilities, and other things in your neighborhood

Please think about one common destination you go to from your house very often, and how many minutes does it take you? Thinking about how long it takes you to walk to this destination can help you with the next questions. *Approximately how long would it take to get from your house to the nearest places or locations listed below if you walked to them? Please put only one check mark (√) for each business or facility.*

	1-5 min	6-10 min	11-20 min	21-30 min	31+ min	Don't know
**example**: gas/petrol station	1. ____	2. ____	3. √___	4. ____	5. ____	8.___
1. kiosk/corner store small grocery	1.____	2. _____	3. ______	4. _____	5. _____	8.__
2. supermarket	1.____	2. _____	3. ______	4. _____	5. ____	8.__
3. fruit/vegetable market (food market)	1.____	2. _____	3. ______	4. _____	5. _____	8.__
4. fast food restaurant	1.____	2. _____	3. ______	4. _____	5. _____	8.__
5. non-fast food restaurant	1.____	2. _____	3. ______	4. _____	5. _____	8.__
6. pub or bar	1.____	2. _____	3. ______	4. _____	5. _____	8.__
7. cinema/theater	1.____	2. _____	3. ______	4. _____	5. ___	8.__
8. place of worship/faith centre(church, mosque, shrine)	1.____	2. _____	3. ______	4. _____	5. ____	8.__
9. computer/cell phone kiosks Places for internet or phone calls	1.____	2. _____	3. ______	4. _____	5. _____	8.__
10. library	1.____	2. _____	3. ______	4. _____	5. _____	8.__
11. Any school	1.____	2. _____	3. ______	4. _____	5. _____	8.__
12. your work place or your school (if a student)	1.____	2. _____	3. ______	4. _____	5. ____	8.__
13. book store (book shop)	1.____	2. _____	3. ______	4. _____	5. ___	8.__
14. health care clinic/hospital	1.____	2. _____	3. ______	4. _____	5. ____	8.__
15. pharmacy/chemist	1.____	2. _____	3. ______	4. _____	5.___	8.__

	1-5 min	6-10 min	11-20 min	21-30 min	31+ min	Don't know
16. salon/barber shop (hair dresser)	1._____	2. ______	3. _______	4. _____	5.___	8.___
17. clothing store (tailoring/fashion/designer shop)	1._____	2. ______	3. _______	4. ______	5.____	8.__
18. electronics shop	1._____	2. ______	3. _______	4. ______	5. ____	8.___
19. public bus or train stop	1._____	2. ______	3. _______	4. ______	5. ____	8.___
20. taxi or motorbike stop	1._____	2. ______	3. _______	4. ______	5.____	8.___
21. sports field or court for basketball, soccer, tennis, etc	1._____	2. ______	3. _______	4. ______	5._____	8.___
22. other outdoor recreation space <u>(park, open space, informal play/recreation area)</u>	1._____	2. ______	3. _______	4. ______	5._____	8.____
23. other indoor recreation facilities <u>(recreation center, gymnasium, health or fitness center)</u>	1._____	2. ______	3. _______	4. ______	5. ____	8.____
24. dance or martial arts classes (karate)	1._____	2. ______	3. _______	4. ______	5. ____	8.___
25. tap/well water, pond, river or stream for fresh water (if plumbing is in house, choose "1-5" minutes)	1._____	2. ______	3. _______	4. ______	5. ___	8.__
26. farm (crop planting/animal herding)	1._____	2. ______	3. _______	4. ______	5. ____	8.___
27. places for hunting/ collecting firewood.	1._____	2. ______	3. _______	4. ______	5. _	8._

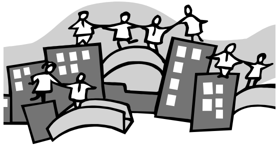

C. Access to services and places

Please circle the answer that best applies to you and your neighborhood. <u>Within easy walking distance</u> means within a 10-15 minute walk from your house.

1. Stores (shops) are within easy walking distance of my house.

1	2	3	4
strongly disagree	somewhat disagree	somewhat agree	strongly agree

2. There are many places to go, such as food markets and restaurants, within easy walking distance of my house.

1	2	3	4
strongly disagree	somewhat disagree	somewhat agree	strongly agree

3. It is easy to walk to a transit/transport stop (bus, taxi, motorbike, tricycle, train) from my house.

1	2	3	4
strongly disagree	somewhat disagree	somewhat agree	strongly agree

4. It is easy to walk to an outdoor recreation play space (park, open space, informal play/recreation area) from my house.

1	2	3	4
strongly disagree	somewhat disagree	somewhat agree	strongly agree

5. It is easy to walk to an indoor recreation facility (recreation center, gym, health or fitness center) from my house.

1	2	3	4
strongly disagree	somewhat disagree	somewhat agree	strongly agree

6. Places to get essential supplies, like water and firewood, are within easy walking distance of my house

1	2	3	4
strongly disagree	somewhat disagree	somewhat agree	strongly agree

7. There are gathering places (community center, king palace, village square, church/worship places etc.) within easy distance of my house.

1	2	3	4
strongly disagree	somewhat disagree	somewhat agree	strongly agree

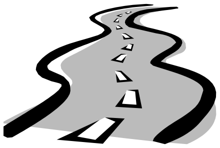

D. Roads and walking paths in my

Please circle the answer that best applies to you and your neighborhood.

1. The distance to walk to the (closest) next street in my neighborhood is usually short (100 meters or less; the length of a football field or less)

1	2	3	4	5
strongly disagree	somewhat disagree	somewhat agree	strongly agree	Not applicable (few official roads)

2. There are many (3 or more) alternative roads (official routes) for getting from place to place in my neighborhood. (I don't have to go the same way every time)

1	2	3	4	5
strongly disagree	somewhat disagree	somewhat agree	strongly agree	Not applicable (few official roads)

3. There are many (3 or more) unofficial routes (walking/foot paths) connecting places in my area.

1	2	3	4
strongly disagree	somewhat disagree	somewhat agree	strongly agree

4. There are many (3 or more) shortcuts such as foot paths between roads (official routes) in my area.

1	2	3	4
strongly disagree	somewhat disagree	somewhat agree	strongly agree

5. Some roads (official routes) or walking/foot paths (unofficial routes) in my area are blocked by gates or barriers.

1	2	3	4
strongly disagree	somewhat disagree	somewhat agree	strongly agree

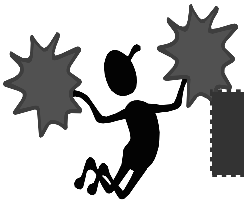

You're making great progress……keep it up!

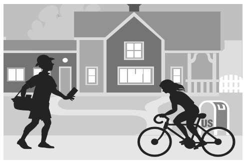

E. Places for walking, cycling and playing

Please circle the answer that best applies to you and your neighborhood.

1. There are formally provided sidewalks (pedestrian pavements) on most of the roads (official routes) in my neighborhood.

1	2	3	4	5
strongly disagree	somewhat disagree	somewhat agree	strongly agree	Not applicable (few official roads)

2. The sidewalks in my neighborhood are well maintained (paved, even, and not a lot of cracks).

1	2	3	4	5
strongly disagree	somewhat disagree	somewhat agree	strongly agree	Not applicable (no sidewalks)

3. The sidewalks in my neighborhood are often blocked by merchandise, construction materials, parked cars, gardens/lawns/barricades.

1	2	3	4	5
strongly disagree	somewhat disagree	somewhat agree	strongly agree	Not applicable (no sidewalks)

4. Sidewalks are separated from the road (vehicle traffic) in my neighborhood by parked cars or dedicated parking bays/curbs.

1	2	3	4	5
strongly disagree	somewhat disagree	somewhat agree	strongly agree	Not applicable (no sidewalks)

5. There is a grass/dirt strip that separates the roads from the sidewalks in my neighborhood.

1	2	3	4	5
strongly disagree	somewhat disagree	somewhat agree	strongly agree	Not applicable (no sidewalks/roads)

6. There are signals or crosswalks/zebra crossings to help walkers cross the busy roads in my neighborhood.

1	2	3	4	5
strongly disagree	somewhat disagree	somewhat agree	strongly agree	Not applicable (few official roads)

7. There are curb ramps (decline or smooth grades) that go from sidewalk level to road level at road crossings (intersections/junctions) in my neighborhood to assist the elderly or wheel chair/prams users

1	2	3	4	5
strongly disagree	somewhat disagree	somewhat agree	strongly agree	Not applicable (no sidewalks)

8. There is enough time for people on foot to cross the road at crossing points/junctions with traffic lights, signals or robots.

1	2	3	4	5
strongly disagree	somewhat disagree	somewhat agree	strongly agree	Not applicable (few official roads)

9. There are informal places (walk/foot paths) for people to walk in my neighborhood.

1	2	3	4	5
strongly disagree	somewhat disagree	somewhat agree	strongly agree	Not applicable (few informal routes)

10. The walk/foot paths in my neighborhood are generally of good quality (few potholes, ditches, un-evenness, stones, obstructions), so it is not difficult to walk there.

1	2	3	4	5
strongly disagree	somewhat disagree	somewhat agree	strongly agree	Not applicable (few informal routes)

11. In my neighborhood/area there are busy roads that are dangerous to cross.

1	2	3	4	5
strongly disagree	somewhat disagree	somewhat agree	strongly agree	Not applicable (few official roads)

12. There are designated or marked places to bicycle, such as separate paths or trails, or shared use paths for cycles and pedestrians in or near my neighborhood.

1	2	3	4
strongly disagree	somewhat disagree	somewhat agree	strongly agree

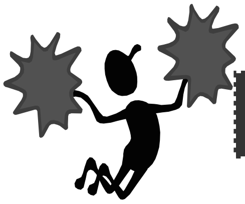

You're making great progress……keep it up!

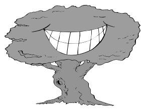

F. Neighborhood surroundings

Please circle the answer that best applies to you and your neighborhood.

1. There are trees along the roads/paths in my neighborhood.

1	2	3	4
strongly disagree	somewhat disagree	somewhat agree	strongly agree

2. My neighborhood is clean and free of litter, garbage or stagnant water.

1	2	3	4
strongly disagree	somewhat disagree	somewhat agree	strongly agree

3. My neighborhood is free from bad smells and odors.

1	2	3	4
strongly disagree	somewhat disagree	somewhat agree	strongly agree

4. There are beautiful natural sights/views in my neighborhood.

1	2	3	4
strongly disagree	somewhat disagree	somewhat agree	strongly agree

5. There are attractive buildings/houses in my neighborhood.

1	2	3	4
strongly disagree	somewhat disagree	somewhat agree	strongly agree

6. My neighborhood is generally free of unpleasant noises like highways, factories, trains, bars, music/record studios, nightclubs/discotheques etc.

1	2	3	4
strongly disagree	somewhat disagree	somewhat agree	strongly agree

7. My neighborhood is generally free of noticeable pollution and dust, such as from traffic or factories.

1	2	3	4
strongly disagree	somewhat disagree	somewhat agree	strongly agree

8. There are many pleasant natural sounds in my neighborhood such as from birds.

1	2	3	4
strongly disagree	somewhat disagree	somewhat agree	strongly agree

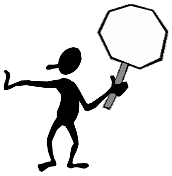

G. Safety from traffic

Please circle the answer that best applies to you and your neighborhood.

1. There is so much traffic along nearby roads that it is difficult or unpleasant to walk or play in my neighborhood.

1	2	3	4	5
strongly disagree	somewhat disagree	somewhat agree	strongly agree	Not applicable (few official roads)

2. The speed of traffic on most nearby roads in my neighbourhood is usually slow

1	2	3	4	5
strongly disagree	somewhat disagree	somewhat agree	strongly agree	Not applicable (few official roads)

3. Most drivers exceed the speed limits (drive very fast) in my neighborhood.

1	2	3	4	5
strongly disagree	somewhat disagree	somewhat agree	strongly agree	Not applicable (few official roads)

4. Walking or playing is dangerous in my neighborhood because of careless or aggressive driving.

1	2	3	4	5
strongly disagree	somewhat disagree	somewhat agree	strongly agree	Not applicable (few official roads)

5. It could be dangerous to ride on a bicycle in or near my neighborhood because of speed of traffic.

1	2	3	4	5
strongly disagree	somewhat disagree	somewhat agree	strongly agree	Not applicable (few official roads)

6. I am worried about playing or walking in my neighborhood and local streets because I am afraid of being injured by a car.

1	2	3	4
strongly disagree	somewhat disagree	somewhat agree	strongly agree

H. Safety from crime

Please circle the answer that best applies to you and your neighborhood.

1. There is a lot of crime in my neighborhood.

1	2	3	4
strongly disagree	somewhat disagree	somewhat agree	strongly agree

2. There is too much crime in my neighborhood to go outside for walks or play during the day.

1	2	3	4
strongly disagree	somewhat disagree	somewhat agree	strongly agree

3. There is too much crime in my neighborhood to go outside for walks or play at night.

1	2	3	4
strongly disagree	somewhat disagree	somewhat agree	strongly agree

4. There are groups of people or gangs (rascals, hooligans, thugs) in my neighborhood who make me feel threatened when I go out.

1	2	3	4
strongly disagree	somewhat disagree	somewhat agree	strongly agree

I. Personal Safety

Please circle the answer that best applies to you and your neighborhood.

1. I see and I can talk to people when I am walking in my neighborhood.

1	2	3	4
strongly disagree	somewhat disagree	somewhat agree	strongly agree

2. There are stray dogs or dangerous animals that scare me in my neighborhood.

1	2	3	4
strongly disagree	somewhat disagree	somewhat agree	strongly agree

3. The roads in my neighborhood are well lit (adequate functioning street lights) at night.

1	2	3	4	5
strongly disagree	somewhat disagree	somewhat agree	strongly agree	Not applicable (few official roads)

J. Stranger Danger

If you are a parent with a child 17 years old or below, please answer the questions below. When responding to the questions, please think mainly about the child who brought the survey.

1. I am worried about letting my child *play or being outside* *alone or with friends* around my house (e.g. yard, driveway, apartment common area), because I am afraid of them being taken or hurt by a stranger.

1	2	3	4
strongly disagree	somewhat disagree	somewhat agree	strongly agree

2. I am worried about letting my child play or walk *alone or with friends* in my neighborhood and local streets because I am afraid of them being taken or hurt by a stranger.

1	2	3	4
strongly disagree	somewhat disagree	somewhat agree	strongly agree

3. I am worried about letting my child be alone or *with friends* in a local or nearby park because I am afraid of them being taken or hurt by a stranger.

1	2	3	4
strongly disagree	somewhat disagree	somewhat agree	strongly agree

If you are 17 years or younger, please answer these questions:

1. I am worried about playing or being outside *alone or with friends* around my house (e.g. yard, driveway, apartment common area), because I am afraid of being taken or hurt by a stranger.

1	2	3	4
strongly disagree	somewhat disagree	somewhat agree	strongly agree

2. I am worried about playing or walking alone or with friends in my neighborhood and local streets because I am afraid of being taken or hurt by a stranger.

1	2	3	4
strongly disagree	somewhat disagree	somewhat agree	strongly agree

3. I am worried about being alone or with friends in a local or nearby park because I am afraid of being taken or hurt by a stranger

1	2	3	4
strongly disagree	somewhat disagree	somewhat agree	strongly agree

THANK YOU AND WE APPRECIATE YOUR HELP IN COMPLETING THE LONG QUESTIONNAIRE
