# Supplementary material for: NEWS for Africa: adaptation and reliability of a built environment questionnaire for physical activity in seven African countries
Source: Int J Behav Nutr Phys Act. 2016 Mar 8;13:33. doi: 10.1186/s12966-016-0357-y (PMC4782343; doi:10.1186/s12966-016-0357-y)
Supplement: Additional file 2: — Summary of results of cognitive testing of NEWS-Africa survey (DOC 42 kb) [file 12966_2016_357_MOESM2_ESM.doc]

**Summary of results from the Cognitive Evaluation of Modified NEWS in 6 African countries**

|  | Sections | South Africa (n=30 adults; middle to low SES areas) | Ghana (n= 6 adults and 6 adolescents; low SES areas) | Kenya (n=30 children; low, middle & high SES areas) | Nigeria (n=4 adolescents and 2 adults; low SES areas) | Cameroon (n=6 adults; low SES areas) | Uganda (n=15 adults and 10 youth; rural & low SES areas) |
| --- | --- | --- | --- | --- | --- | --- | --- |
| 1 | Demographics (12 items) | Question on income was difficult to answer | Section was Okay | Questions on weight, height, income, marital status, age of household members were difficult. | Income, height, weight were difficult to recall by adolescents | Question on height, weight and how many adults live in household were difficult. Suggest including occupation | Some could not answer on their age, response options to question on usage of private vehicle not clear, and height and weight difficult |
| 2 | Types of residence (single item, 6 response options) | Difficult to understand | Okay | Okay | Some difficulties to independently cue the housing types by the adolescents | Okay | Many respondents could not link 6 stories building with multiple apartment blocks |
| 3 | Store, facilities (27-items) | 4 participants find it difficult to understand distance and time taken to walk to destinations | Recommend “not applicable” response option for 12- items | Okay but few children unsure of time taken to move from place to place | Okay | 6-items required modifications to be applicable in Cameroon | Difficult to estimate distance by some respondents. Recommend adding ‘Not any’ option to the response scale. |
| 4 | Access to services & places  (7-items) | Okay | Okay | The most difficult section for the participants. The response options not easy to understand by children | Difficult to independently handle the response options by adolescents | Okay | The word “it is easy” was found ambiguous and difficult to understand by many participants |
| 5 | Roads and walking paths  (5-items) | Items were difficult to answer by 3 participants | Okay | Items not clear to respondents from low SES but good for middle and high SES respondents | Okay | Okay | Recommend clarifying what “many” is and suggested putting three or more in parentheses. |
| 6 | Places for walking, cycling & playing (13-items) | Okay | Okay | Items not clear to respondents from low SES but good for middle and high SES respondents | 5-items that focused on rare pedestrian infrastructures were not clear to the adolescents | Okay | Some respondents were not cleared if designated places to bicycle applied to all roads or some roads |
| 7 | Neighbourhood surrounding (8-items) | Okay | Okay | Items not clear to respondents from low SES but good for middle and high SES respondents | Okay | Okay | Okay but suggested deleting the word “many” from the item on beautiful natural sights and views |
| 8 | Safety from traffic (5-items) | Okay | Okay | Okay | Okay | Okay | Okay with some edits |
| 9 | Safety from crime (4- items) | Okay | Okay | Okay | Okay | Suggest inclusion of one additional item (vigilante groups) | Okay |
| 10 | Child’s Questions (4-items) | Okay | Okay | Okay | Okay | Okay | Okay with some edits |
| 11 | Additional items (2-items) | Okay | Okay | Okay | Okay | The two items were ambiguous | Okay |
