# Supplementary material for: NEWS for Africa: adaptation and reliability of a built environment questionnaire for physical activity in seven African countries
Source: Int J Behav Nutr Phys Act. 2016 Mar 8;13:33. doi: 10.1186/s12966-016-0357-y (PMC4782343; doi:10.1186/s12966-016-0357-y)
Supplement: Additional file 3: — Interview manual for NEWS-Africa survey (PDF 375 kb) [file 12966_2016_357_MOESM3_ESM.pdf]

INTERVIEW MANUAL FOR NEWS-AFRICA SURVEY

As an interviewer administering the NEWS-Africa survey, you are required to be familiar with the information in this manual. The information is to help inform you about the meaning of items in the survey and guide you on what and how to prompt and explain if some questions are difficult for the respondents. Please ensure you understand and clarify with the research team all difficult items not clear to you before proceeding with respondents' interviews.

SECTION A: DEMOGRAPHIC INFORMATION

The demographic section of NEWS-Africa survey was designed to collect demographic variables that are comparable across African countries. When using NEWS-Africa it is important to collect demographic data in ways that are consistent with those described below:

**Variables**	**Indicators**
1. Age:	Ask for the age in years or the date of birth from the respondents
2. Sex:	Collect data to be able to determine % of female from total sample
3. Marital status:	Collect data to be able to dichotomize into "Married/living with partner" vs "others". Mark not "not applicable" for youth
4. Education:	Collect data to allow for grouping into "less than high school", "some high school/completed high school", and "more than high school"
5. Motorized transport:	"None", "one", "two", and "three or more" are required indicators for this variable
6. Frequency of motor:	Less than once a week (including none), a few times a week, most days of the week, and everyday are the minimum required indicators for this variable
7. Income:	Not a compulsory variable for NEWS-Africa, but if possible to collect from the respondents, the idea is to identify three categories ("low income", "middle income" and "high income"), so use the cut-points specified by the research team (Note that this variable is not relevant for youth)
8. Adults in household:	Count adults who live there "most of the time"
9. Youth in household:	Count youth 17 years or younger who live there "most of the time"
10. Present address:	Collect either the specific street address with city/town, or nearest street intersection

11. Height: Collect data as continuous variable (direct measurement or self-report). Specify the unit in metres

12. Weight: Collect data as continuous variable (direct measurement or self-report). Specify the unit in kg

SECTION B: QUESTION ABOUT YOUR NEIGHBOURHOOD

Instruction: Please emphasize to the respondents that the intention of this section is to know more about the areas where they live, and their perceptions about the built environment (houses, roads, infrastructures, and physical and social facilities) in their neighbourhood as it affects their mobility and activities such as walking, cycling and playing. Please, ***emphasize to the respondents to think of their neighbourhood as all the areas that are about one kilometer or half a mile of their house or all the areas that they can walk to in 10-15 minutes*.**

A- TYPES OF HOUSING

The intention of this section is to capture the housing density in the areas where the respondent lives. Please explain to the respondents that the various housing types have been ordered and ranked from the lowest (1) to the highest (6) residential density. Please use the provided photos (#1 to #6) of the various housing types to aid the respondents' understanding of the main type of housing in their neighbourhood. Respondents can only select one type of housing in this section.

B- STORE, FACILITIES, AND OTHER THINGS IN YOUR NEIGHBOURHOOD

The purpose of this section is to rate the time it takes to walk to diverse places, destinations or locations from the respondent's home. Note that the various places or destinations listed in the questionnaire are not necessarily intended to be available in the respondent's immediate neighbourhood, but at any nearest locations in or outside of their neighbourhood. Respondents do not need to estimate the exact minutes; they can use the ranges of minutes on the questionnaire.

For participants having difficulties estimating the time taken to walk to any of the listed destinations, please prompt them to estimate this time relative to the time taken to walk to a common destination they always go (e.g., faith based places, work or school). This can help them estimate distances to walk to places that are nearer and farther. "Walking" refers to their usual pace or speed.

If participants do not know where a destination is (absent or not available in the neighbourhood) or it takes longer than one hour to go, please instruct them to choose the "Don't know" option or to answer with the highest category of minutes on the response scale.

C- <u>ACCESS TO SERVICES AND PLACES</u>
This section focuses on the degree to which respondents can access important places and destinations in their neighbourhood within easy walking distance from homes. ***Please clarify to respondents that the words "It is easy to walk" and "Easy walking distance" used in this section can mean 10-15 minutes.*** For each of the items, please emphasize only the examples that are specific to the respondents' location rather than all the listed examples. For example, when interviewing respondents who live in the city, it will be more appropriate and less confusing to prompt them about gathering places such as faith places (e.g., church, mosque) rather than on king palace, village square and community center.

To enhance the understanding of the scoring of items in these section and the remaining sections, please prompt and carefully explain the response options to the respondents. For example, ask the respondents to first indicate whether they agree or disagree to the question. Then follow-up and ask whether the degree of agreement/disagreement is strong or somewhat (a bit). Please use these definitions as guide:

"strongly agree" means to say **"definitely yes"** to the question
"strongly disagree" means to say **"definitely no"** to the question
"somewhat disagree" means to say **"a bit or little no"** to the question
"somewhat agree" means to say **"a bit or little yes"** to the question

D- <u>ROADS AND WALKING PATHS IN MY NEIGHBOURHOODS</u>
The purpose of the questions in this section is to assess the perceptions of the respondents on how connected the roads or streets in their neighbourhoods are, and to know if it is easy to move (walk or bicycle) from one location to the other in their neighbourhood without barriers. Please note that a well-connected street will have more intersections (closer to many other streets), more shortcuts and many alternative roads for getting from place to place.

To improve the clarity of items in this section, please emphasize and explain the distinction between road and pathways/shortcuts/foot paths to the respondents. Roads are formal routes (either tarmacked or not), that is, official places for cars with or without pedestrian facilities. On the other hands, pathways, shortcuts and foot paths are informal (unofficial) places where people can walk or bicycle but not primarily meant for cars. Please use photos where necessary to facilitate better understanding of these concepts and questions among the respondents. Photos #7 provide examples of formal/official routes (roads), while photos #8 provide examples of informal/unofficial routes (pathways, footpaths, shortcuts).

E- <u>PLACES FOR WALKING, CYCLING AND PLAYING</u>
The aims of this section are to assess the availability and quality of pedestrian infrastructures/ facilities in the respondents' neighbourhood, and to gauge their perceptions on safety of places they walk, cycle and play. In addition to probing to improve the clarity of the response options and concepts of formal (roads) and informal (walk paths) routes as indicated in section C and D above, please use the provided photos (#9 through #15) to facilitate the understanding of each of items Q1 (sidewalks), Q4 (sidewalks separated from road by parked cars or dedicated parking bay/curbs), Q5 (sidewalks separated from roads by grass/dirt strip), Q6 (signals or

crosswalks/zebra crossings), Q7 (curb ramps that from sidewalk to road level at road crossings), Q8 (cross points/junctions with traffic lights, signals or robots), and Q12 (separate paths or trails or shared used paths for bicycling). For Q12, please clarify to the respondents that the assumption of marked places is not for all routes but some routes in their neighbourhoods.

F- NEIGHBOURING SURROUNDINGS

Questions in this section are to assess the aesthetic qualities of neighbourhood surroundings. As a guide, an aesthetically good neighbourhood will not only be pleasant and imbued with beautiful and attractive features but also be clean and free of litter, garbage, odor, noise and other pollution. One strategy that can be used to improve respondents' responses to aesthetic items is to first ask the participants to indicate whether they agree or disagree with each of the statements while thinking aloud, then have them to rate the degree of their agreement or disagreement using the method cited in section C above.

G, H & I – SAFETY FROM TRAFFIC AND CRIME, AND PERSONAL SAFETY

The purpose of these three sections is to assess the residents' perceptions on how speed of traffic and crime rate, and concern about their personal safety affect their ability and willingness to walk and cycle in their neighbourhood. Please use some of the strategies highlighted in sections C, D., E and F above to improve respondents' responses to the items in these two sections.

J – STRANGER DANGER

Please ensure that the parents or guardians think about the child who brought home the survey when responding to questions in this section.
