# Supplementary material for: NEWS for Africa: adaptation and reliability of a built environment questionnaire for physical activity in seven African countries
Source: Int J Behav Nutr Phys Act. 2016 Mar 8;13:33. doi: 10.1186/s12966-016-0357-y (PMC4782343; doi:10.1186/s12966-016-0357-y)

A: Types of Residences in your Neighbourhood

Photo #1- (A1: Very Few Residential Buildings/Dwellings within 2-5 Minutes Walk)

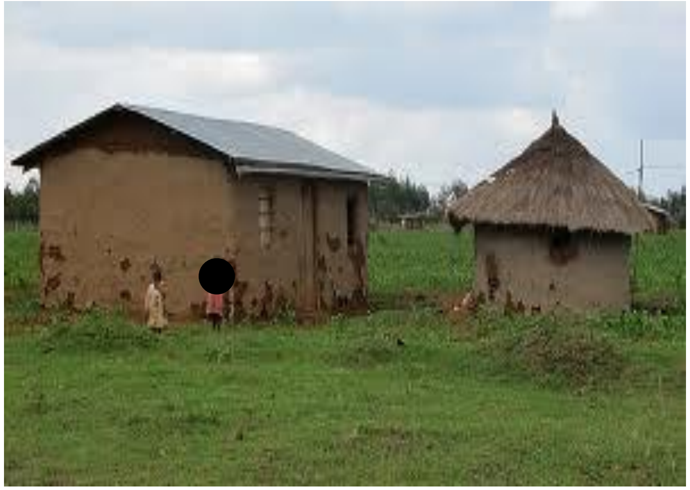	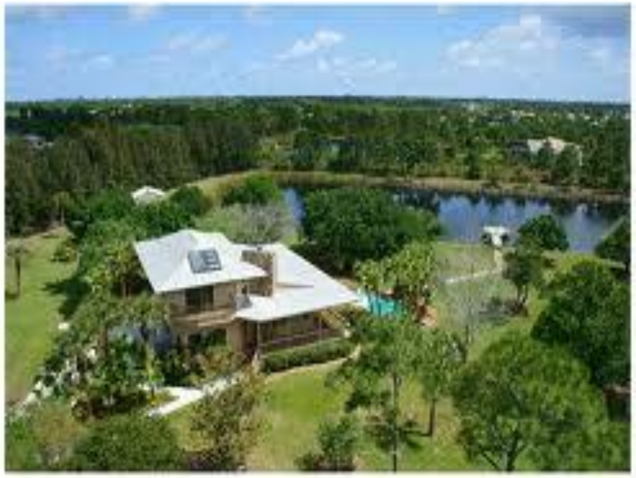
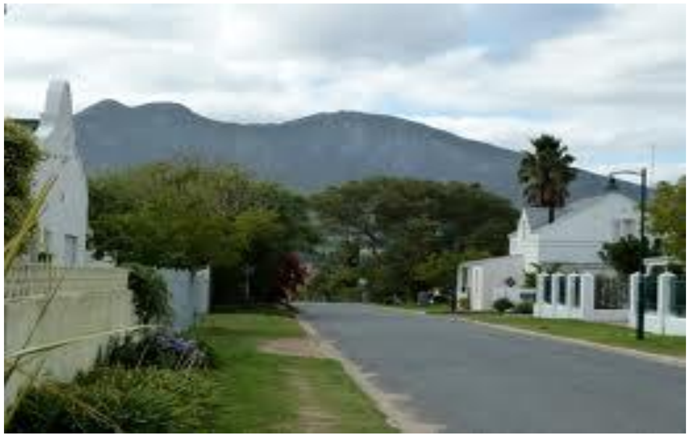	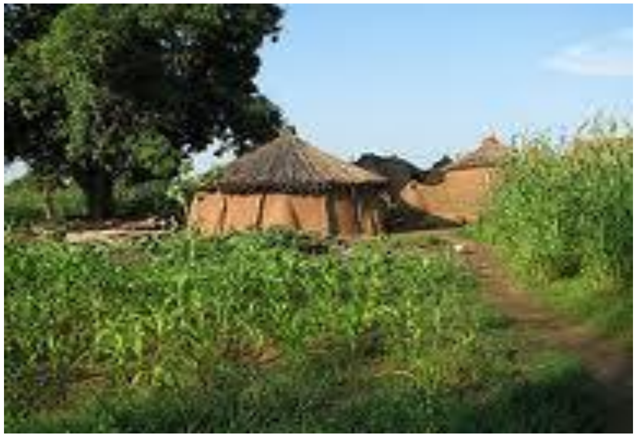

Photo #2- (A2: Detached or Semi-Detached Single-Family houses with Space/Garden)

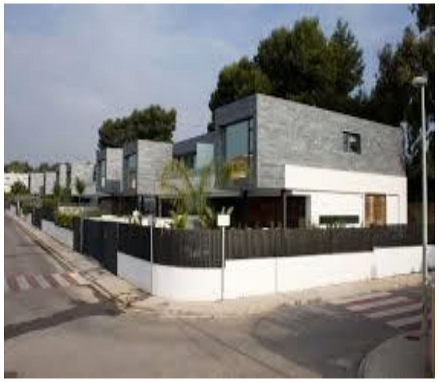
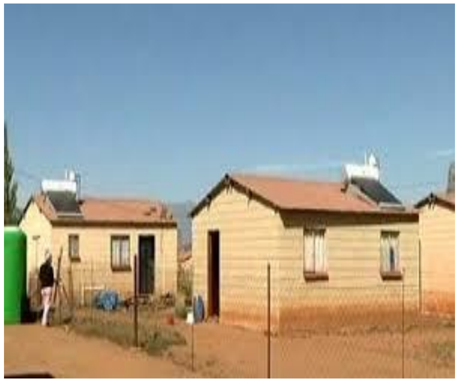
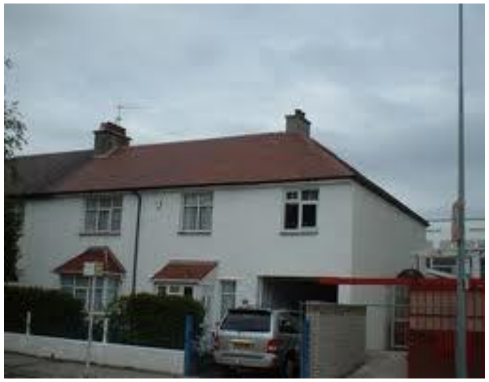
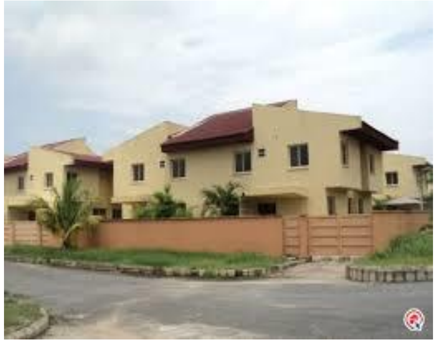
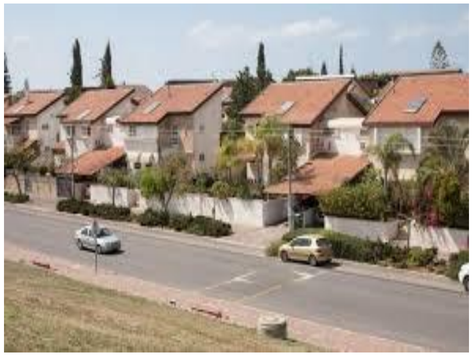
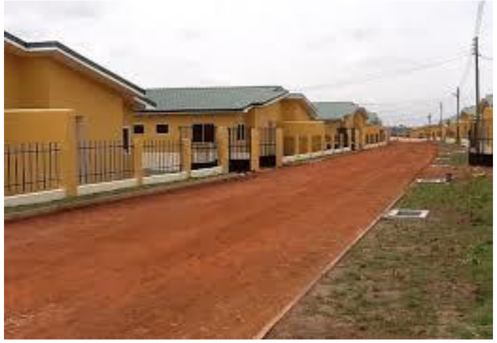

Photo #3- (A3: Attached (Row) Housing, Apartment Block/Flats or multiple family housing with 2-5 stories)

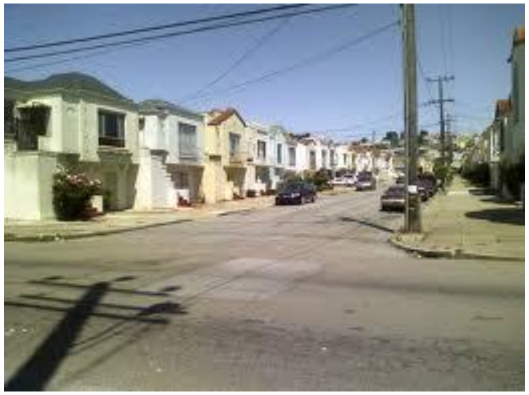

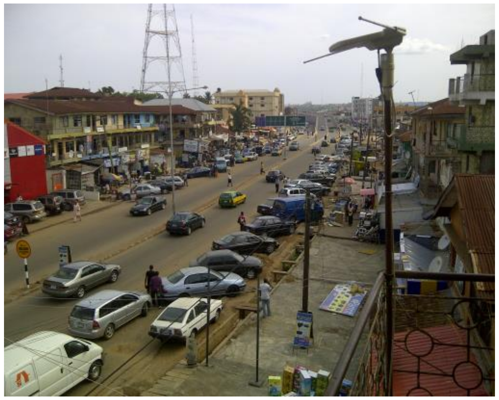

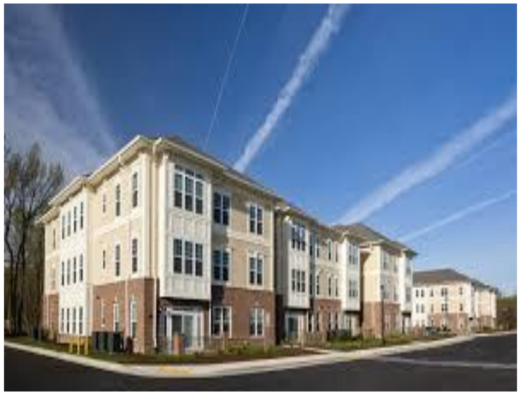

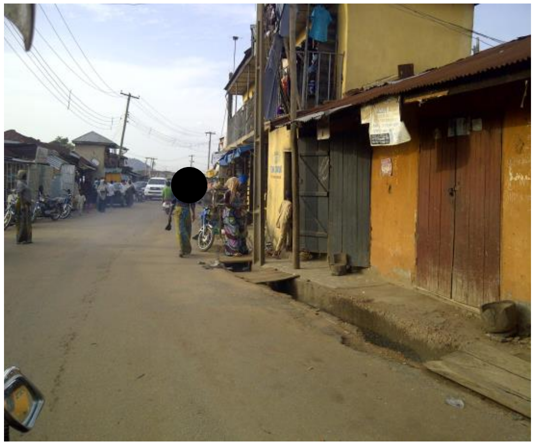

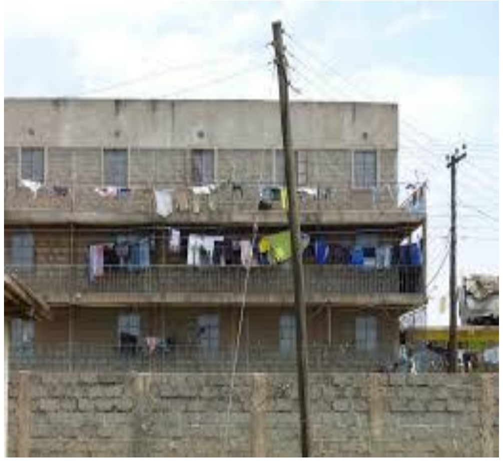

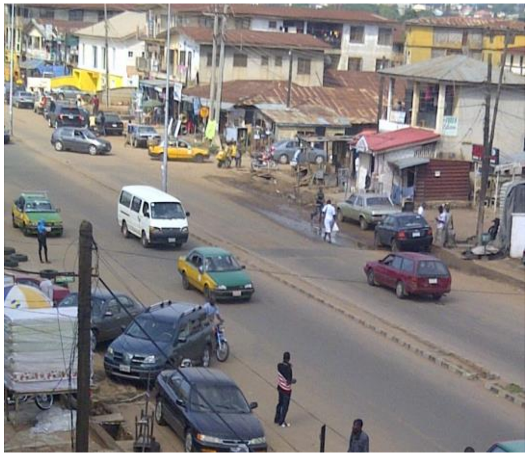

Photo #4- (A4: Multiple Apartment Blocks/Flats of 6 Stories or More, with Large Spaces between Buildings)

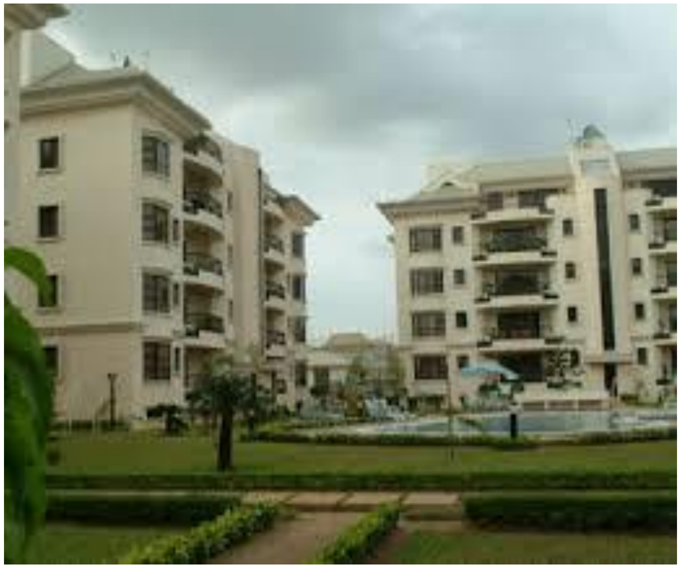

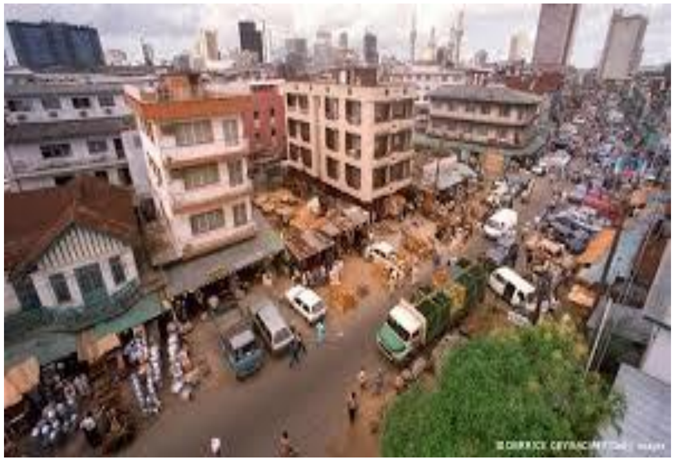

Photo #5- (A5: Multiple Apartment Blocks/Flats of 6 Stories or More, With Very Little Space Between Buildings)

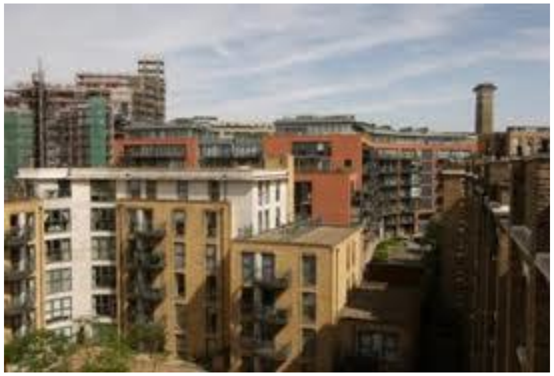

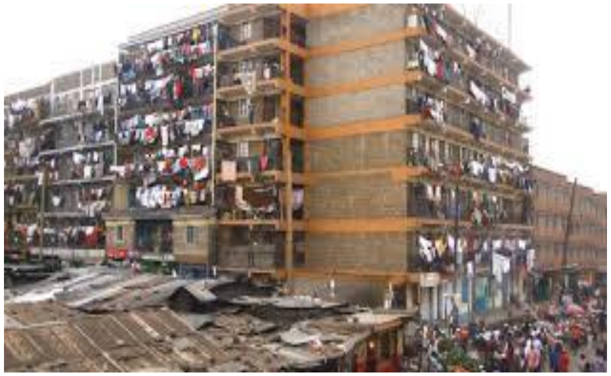

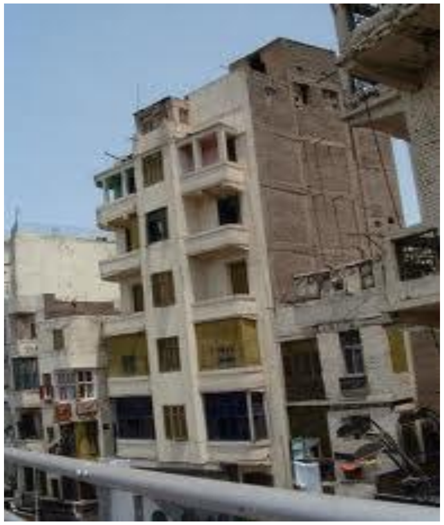

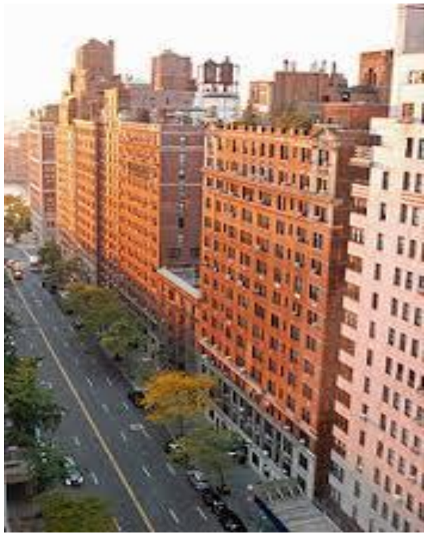

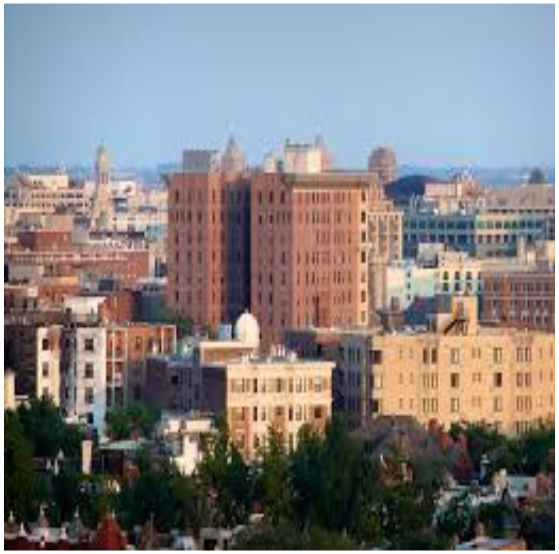

Photo #6- (A6: Very Densely Packed Small Houses (1-Story Homes, including Informal Settlements and Slums))

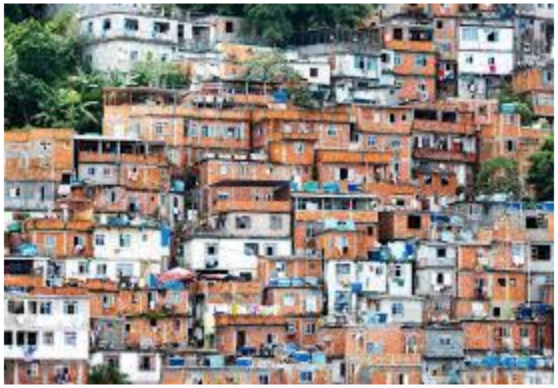

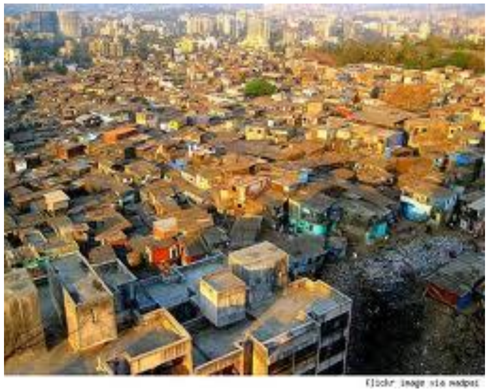

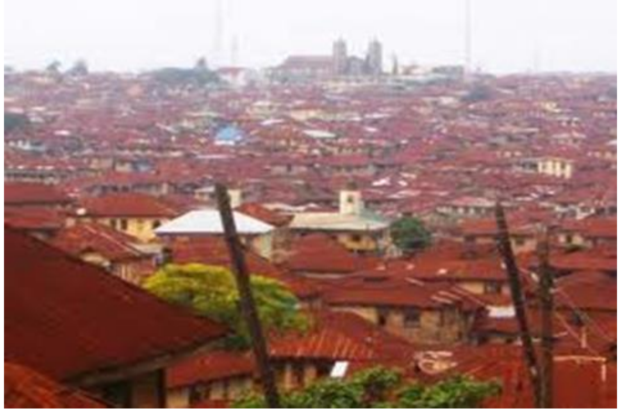

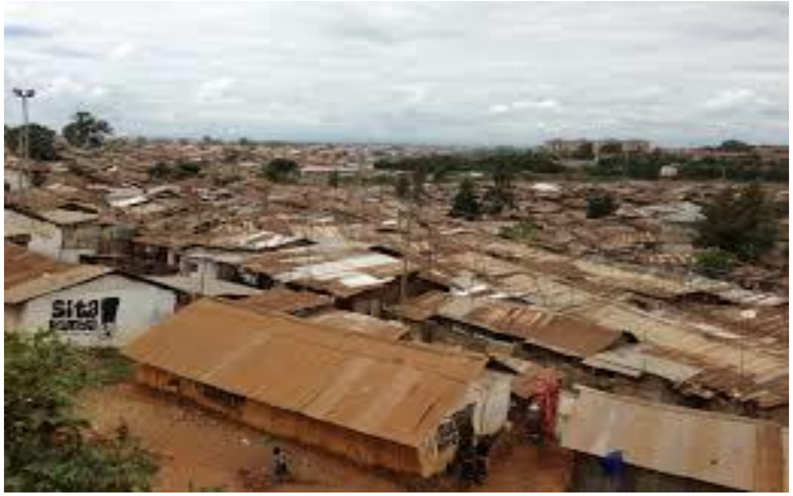

D: Road and Walking Paths in the Neighbourhood

Photo #7- (D1-5: Formal/Official Route (e.g Roads))

Tarmacked Roads	Non- Tarmacked Roads
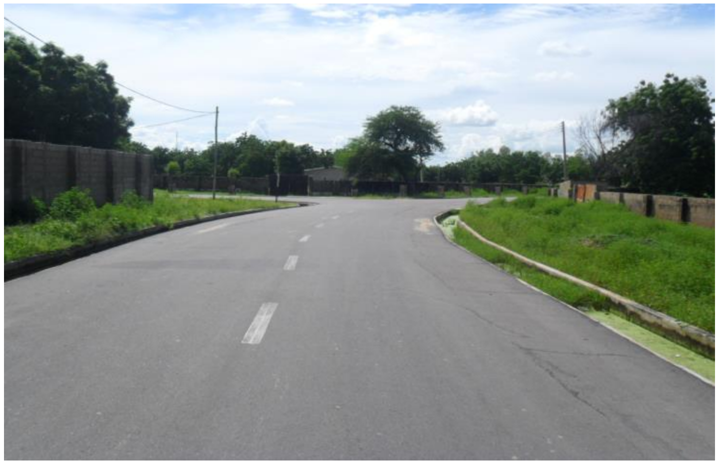	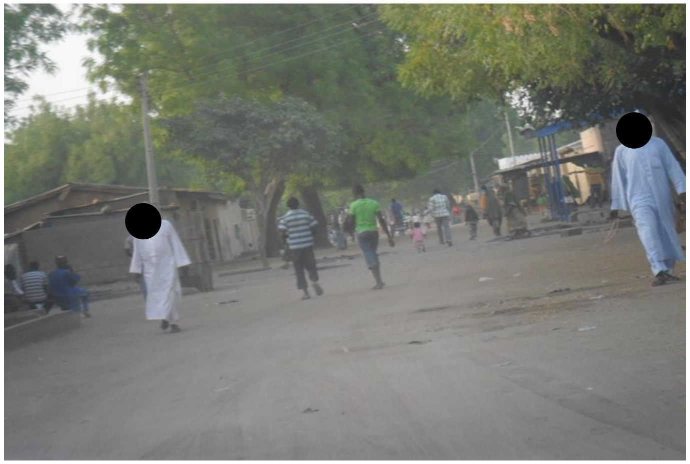
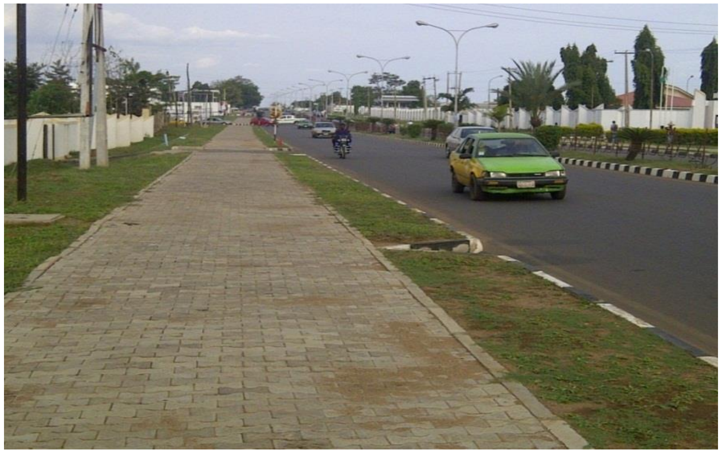	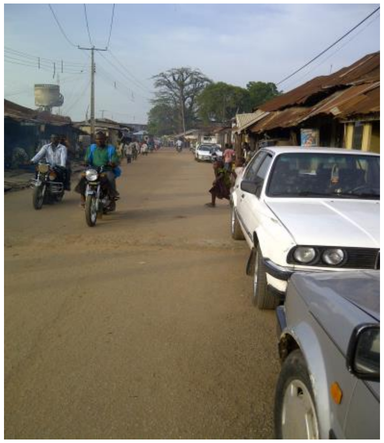 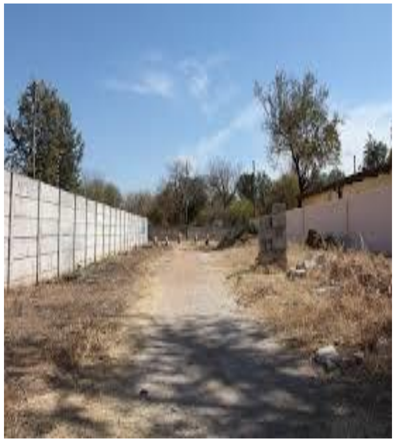

Photo #8- (D1-5: Informal/Unofficial Route (e.g Walk paths, foot paths, pathways etc))

<table>
<tr>
<td>Pathway/footpath beside a Road
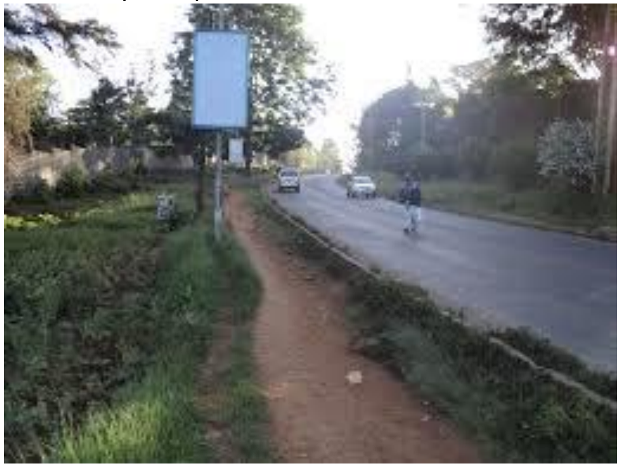</td>
<td>Pathways/footpath in a village
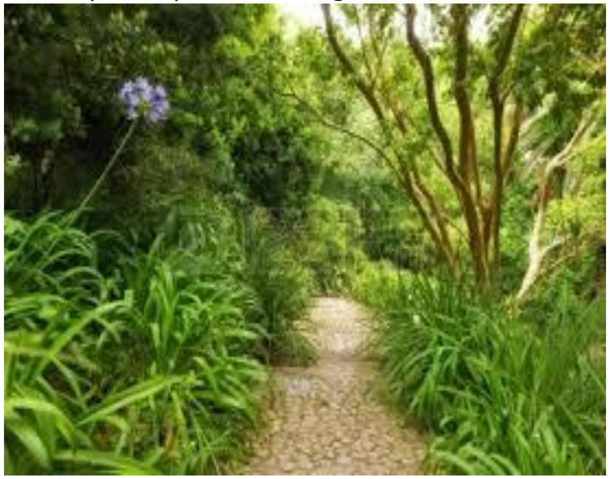</td>
</tr>
<tr>
<td>Pathway/footpath in a street
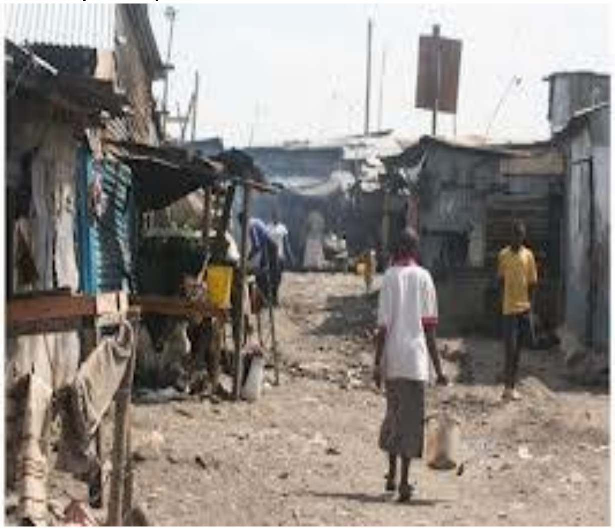</td>
<td>Pathway/footpath beside a road
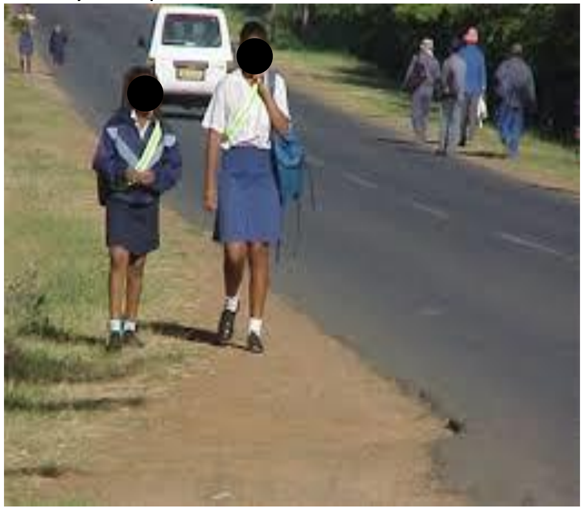</td>
</tr>
</table>

E: Places for walking, cycling and playing

Photo #9 (E1- Sidewalks)

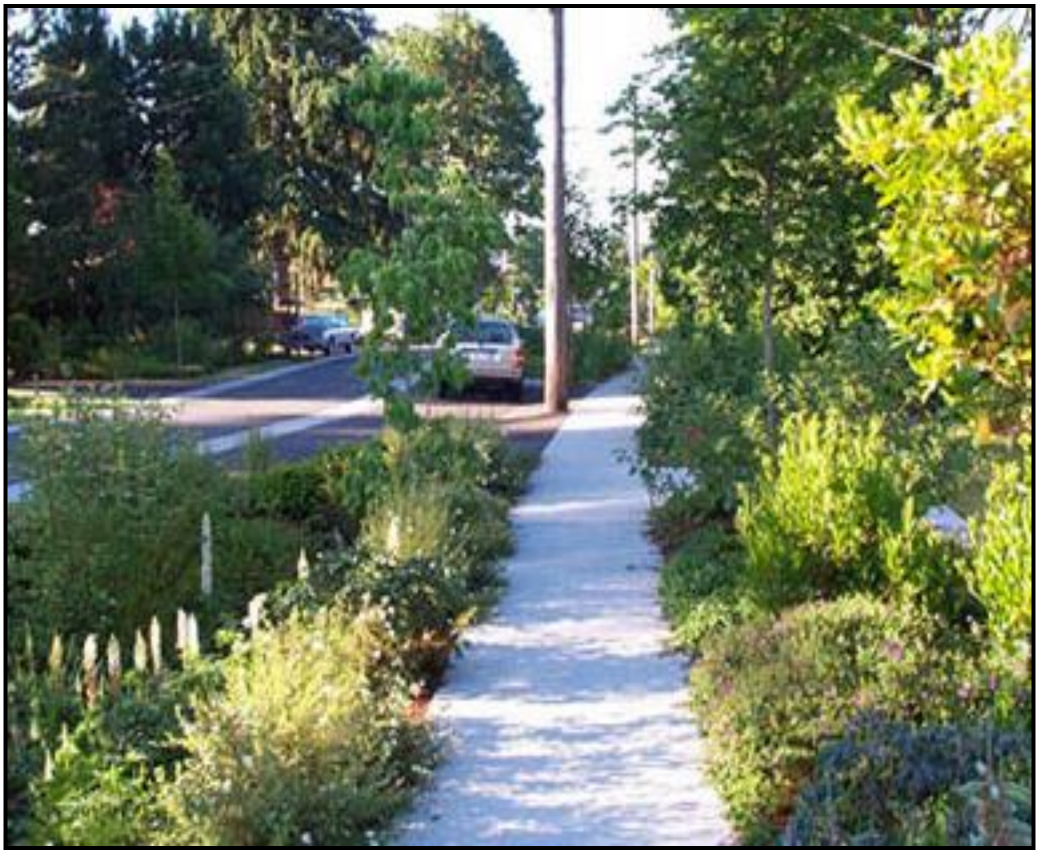

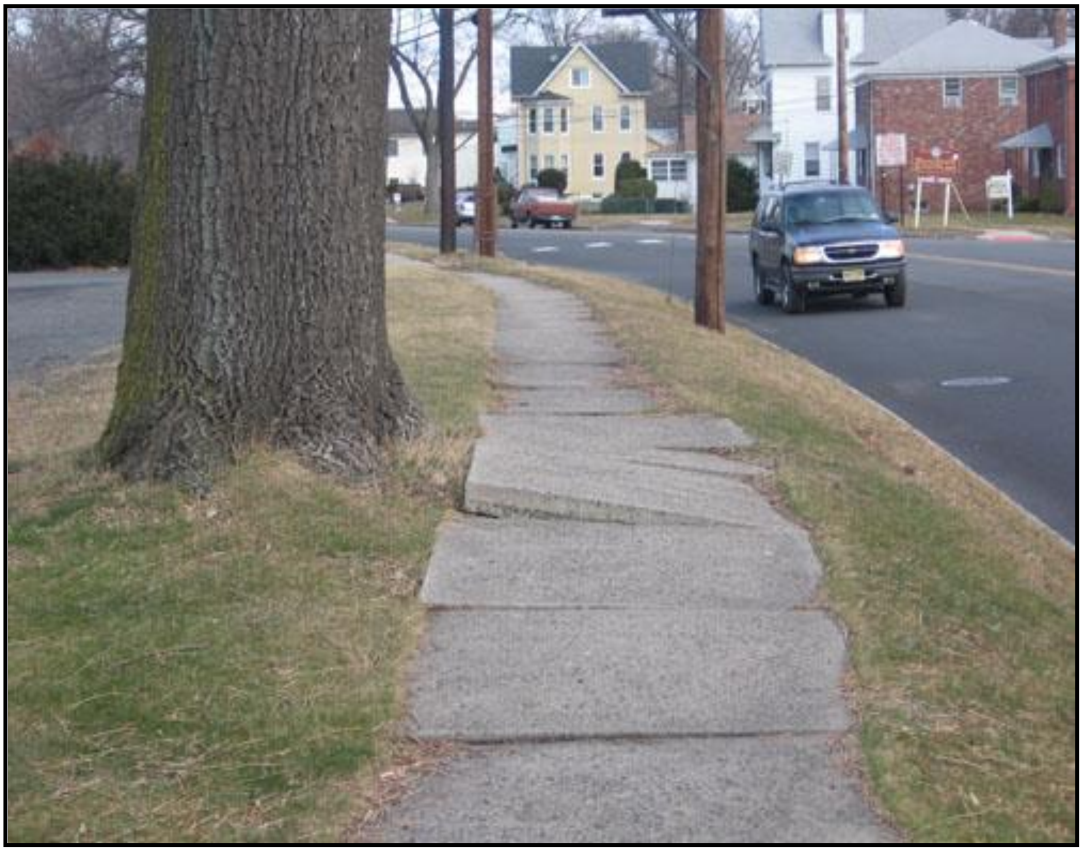

Photo #10 (E4-sidewalks separated from road by parked cars or dedicated parking bay/curbs),

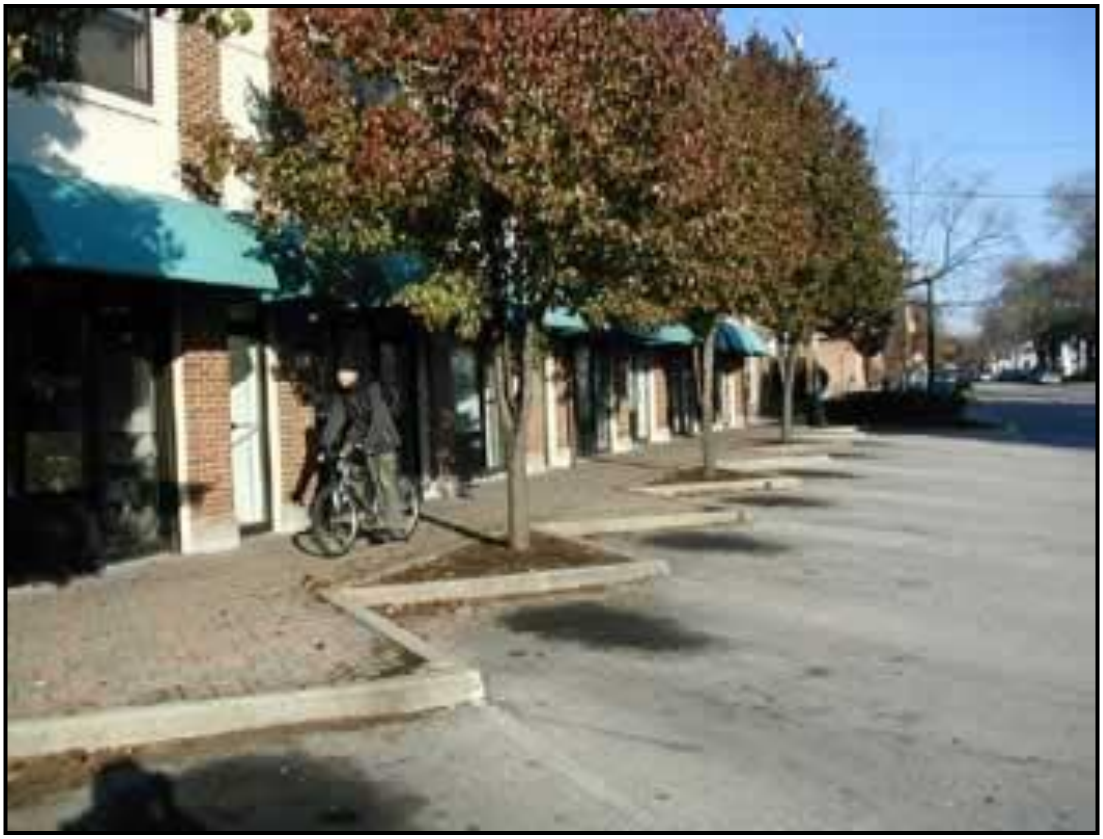

Photo #11 (E5- sidewalks separated from roads by grass/dirt strip),

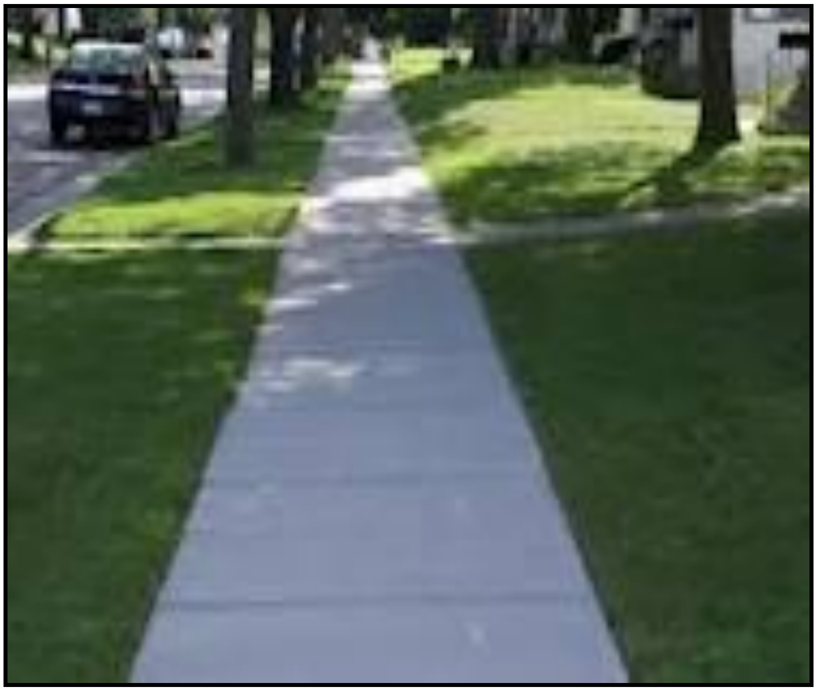

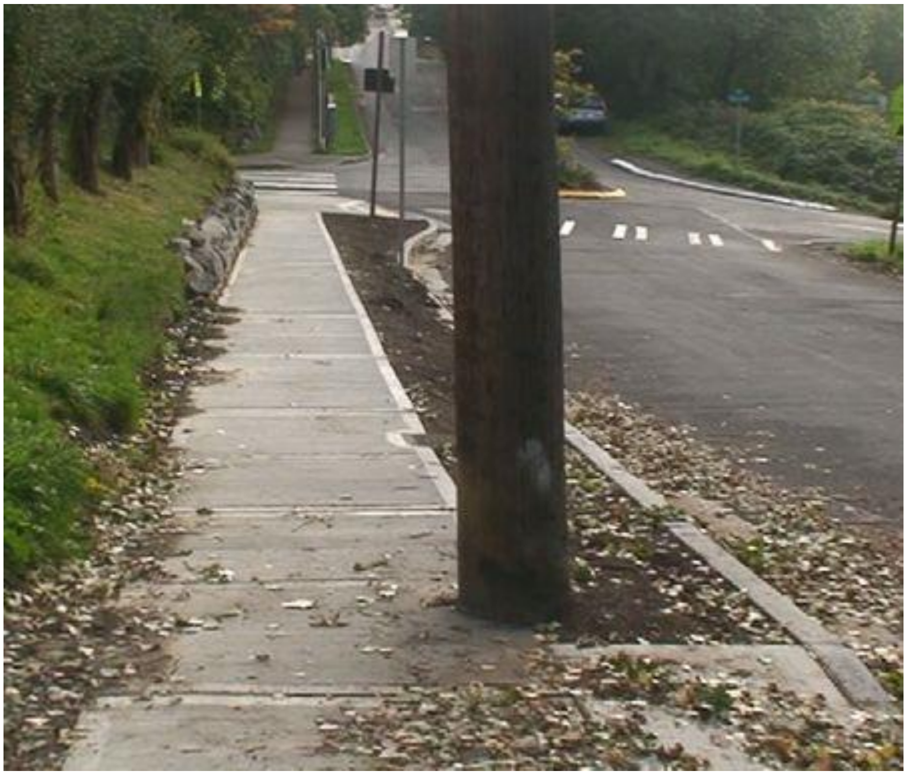

Photo #12 (E6-Signals or crosswalks/zebra crossings),

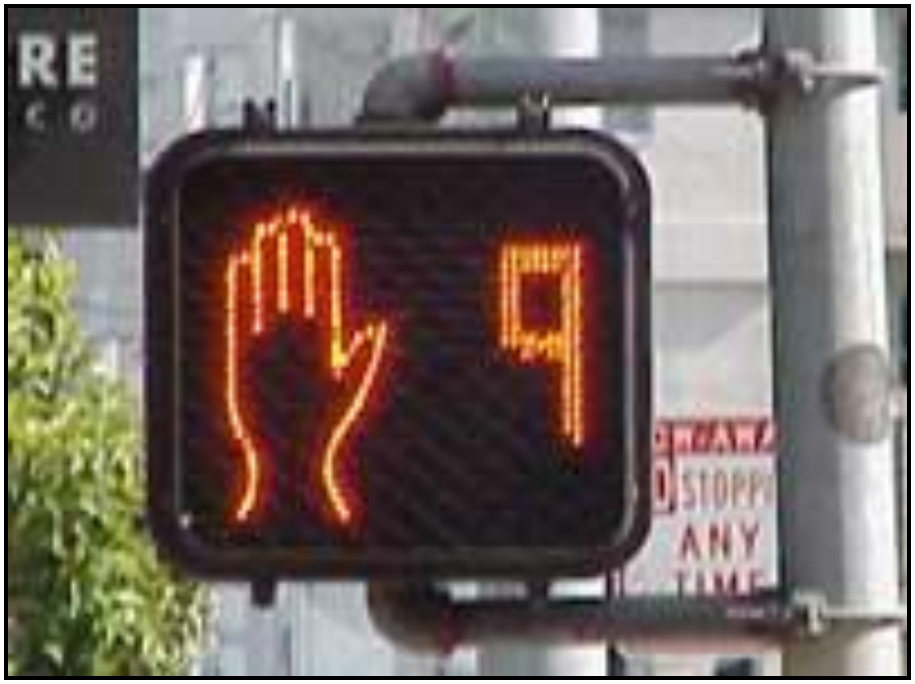

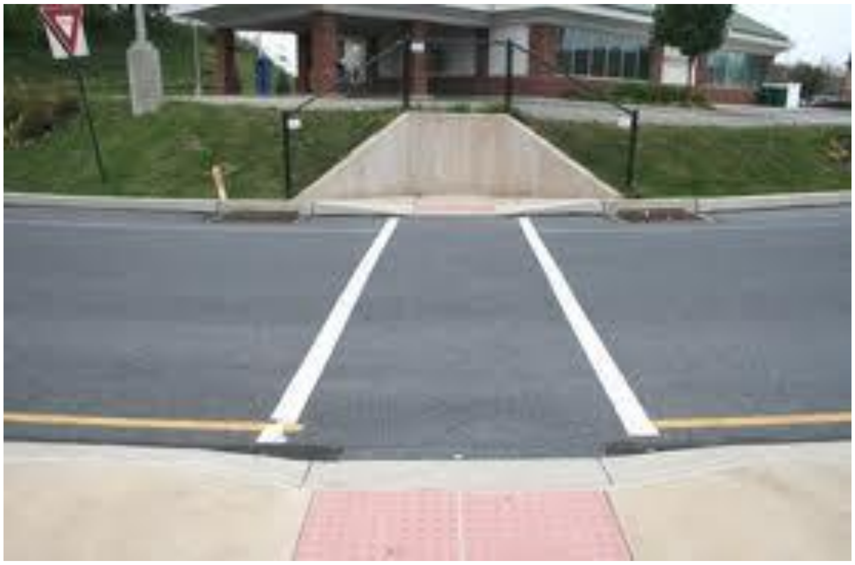

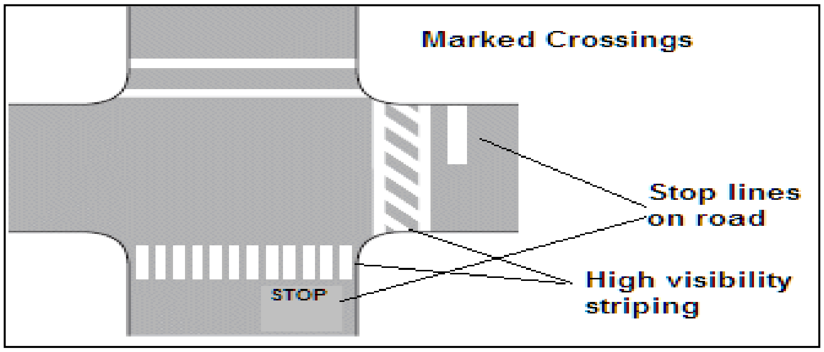

Photo #13 (E7-Curb ramps that from sidewalk to road level at road crossings),

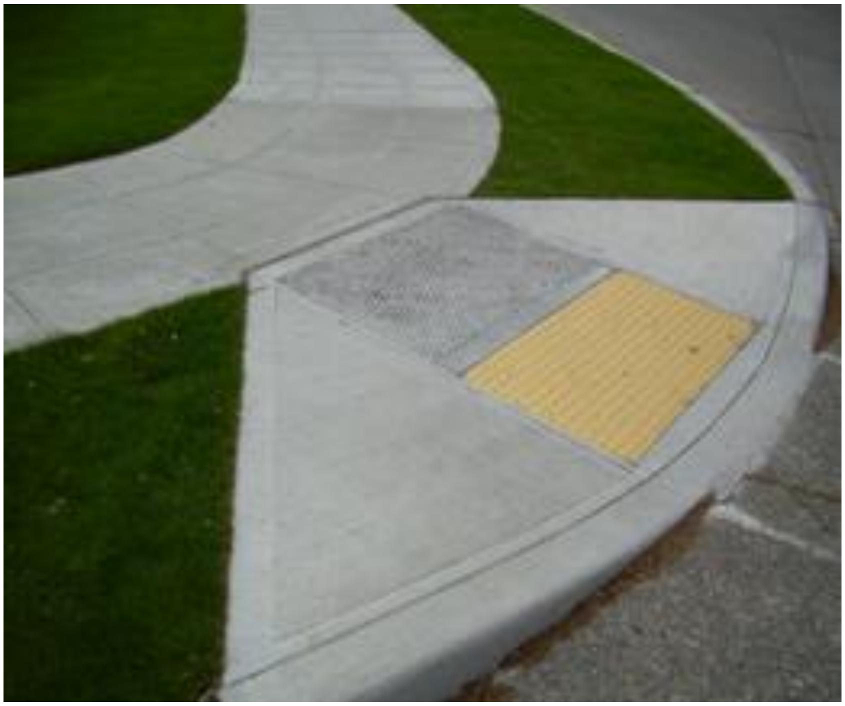

Photo #14 (E8- Cross points/junctions with traffic lights, signals or robots)

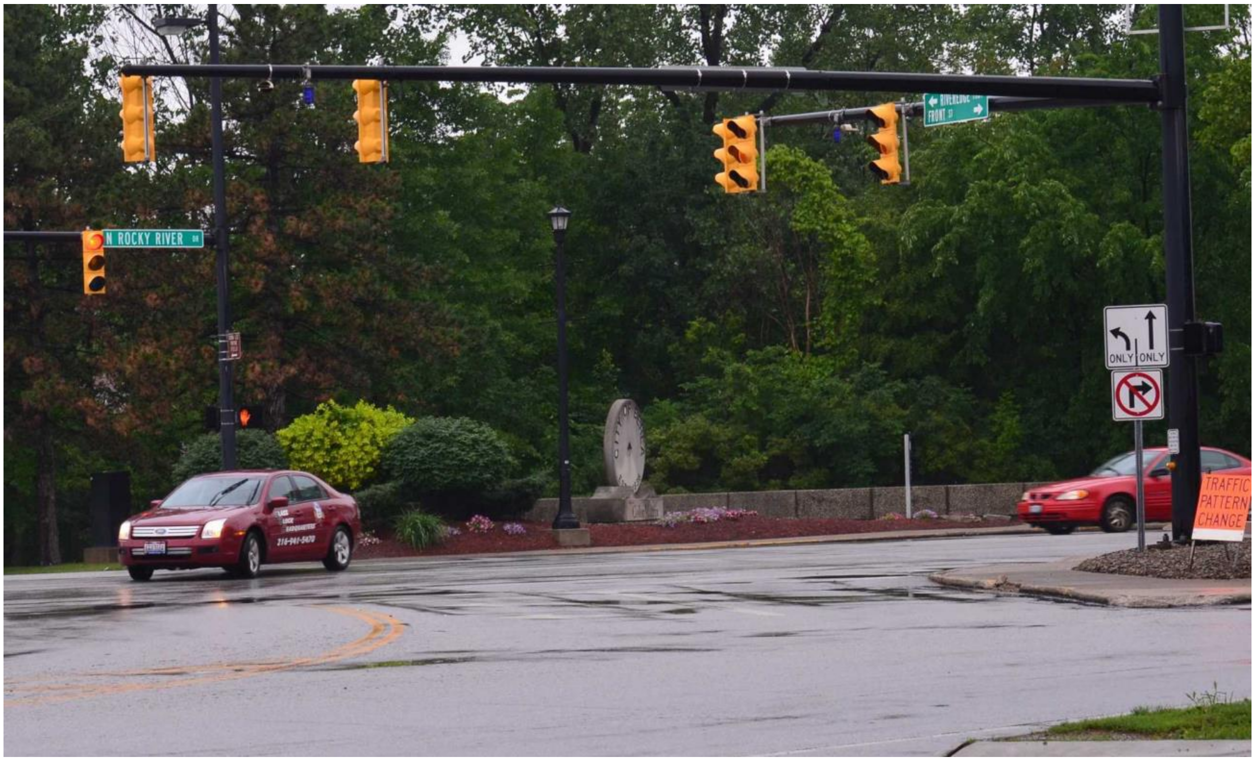

Photo #15 (E13- Separate paths or trails or shared used paths for bicycling)

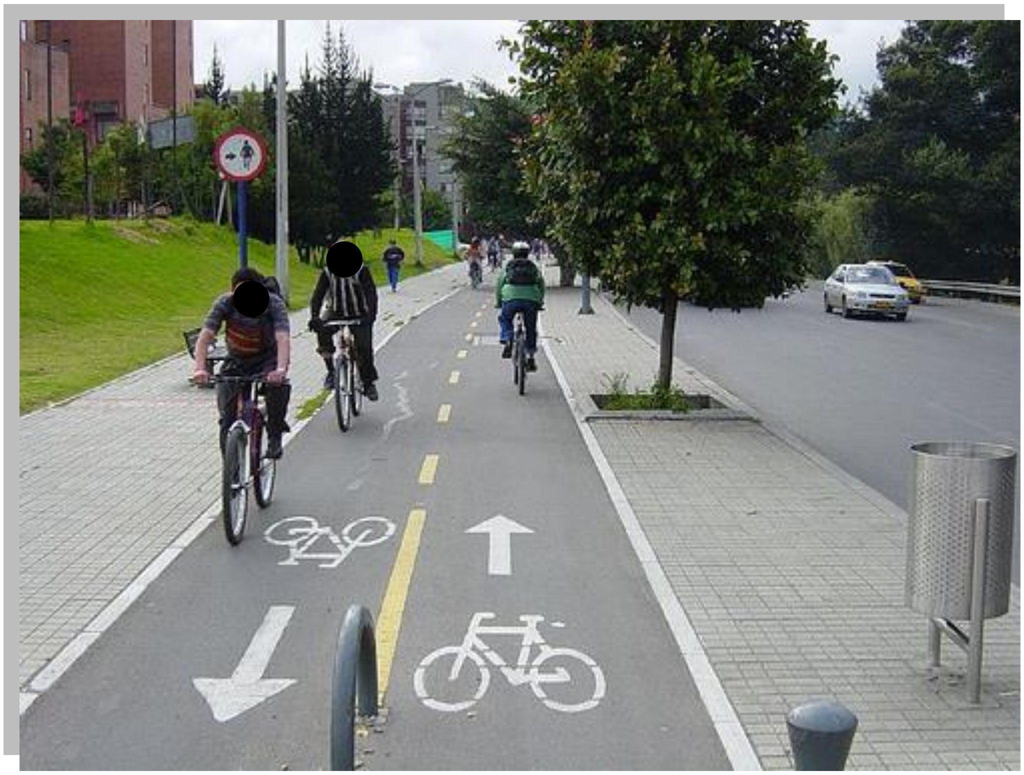

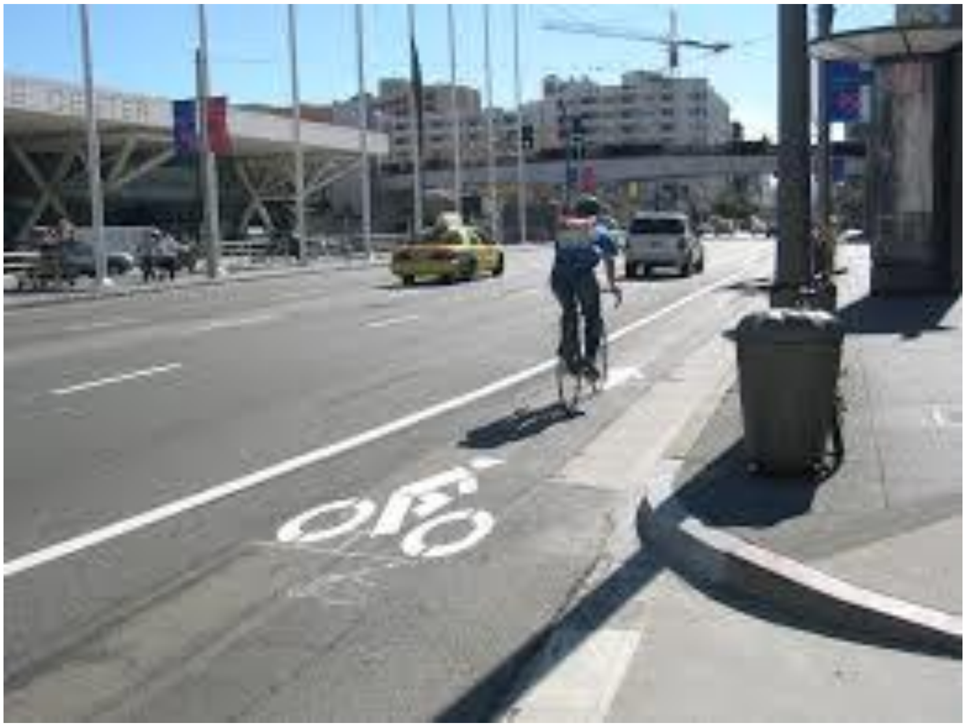

Supplement: Additional file 4: — Picture guide for NEWS-Africa survey (PDF 1318 kb) [file 12966_2016_357_MOESM4_ESM.pdf]
